# Inhibition mechanism of alpha-amylase, a diabetes target, by a steroidal pregnane and pregnane glycosides derived from *Gongronema latifolium* Benth

**DOI:** 10.3389/fmolb.2022.866719

**Published:** 2022-08-10

**Authors:** Oludare M. Ogunyemi, Gideon A. Gyebi, Afolabi Saheed, Jesse Paul, Victoria Nwaneri-Chidozie, Olufunke Olorundare, Joseph Adebayo, Mamoru Koketsu, Nada Aljarba, Saad Alkahtani, Gaber El-Saber Batiha, Charles O. Olaiya

**Affiliations:** ^1^ Human Nutraceuticals and Bioinformatics Research Unit, Department of Biochemistry, Salem University, Lokoja, Nigeria; ^2^ Nutritional and Industrial Biochemistry Unit, Department of Biochemistry, University of Ibadan, Ibadan, Nigeria; ^3^ Department of Biochemistry, Faculty of Science and Technology Bingham University, Nasarawa, Nigeria; ^4^ Natural Products and Structural (Bio-Chem)-informatics Research Laboratory (NpsBC-Rl), Bingham University, Nasarawa, Nigeria; ^5^ Faculty of Basic Medical Sciences, Department of Pharmacology and Therapeutics, Faculty of Basic Clinical Sciences, University of Ilorin, Ilorin, Nigeria; ^6^ Department of Biochemistry, Faculty of Life Sciences, University of Ilorin, Ilorin, Nigeria; ^7^ Faculty of Engineering, Department of Chemistry and Biomolecular Science, Gifu University, Gifu, Japan; ^8^ Department of Biology, College of Science, Princess Nourah Bint Abdulrahman University, Riyadh, Saudi Arabia; ^9^ Department of Zoology, College of Science, King Saud University, Riyadh, Saudi Arabia; ^10^ Department of Pharmacology and Therapeutics, Faculty of Veterinary Medicine, Damanhour University, Damanhour, Egypt

**Keywords:** diabetes, alpha-amylase, *G. latifolium*, phytochemicals, pregnanes, molecular docking, molecular dynamics simulations

## Abstract

Alpha-amylase is widely exploited as a drug target for preventing postprandial hyperglycemia in diabetes and other metabolic diseases. Inhibition of this enzyme by plant-derived pregnanes is not fully understood. Herein, we used *in vitro*, *in silico*, and *in vivo* studies to provide further insights into the alpha-amylase inhibitory potential of selected pregnane-rich chromatographic fractions and four steroidal pregnane phytochemicals (SPPs), viz: marsectohexol (P1), 3-*O*-[6-deoxy-3-*O*-methyl-β-D-allopyranosyl-(1→14)-β-D-oleandropyranosyl]-11,12-di-*O*-tigloyl-17β-marsdenin (P2), 3-O-[6-deoxy-3-O-methyl-β-D-allopyranosyl-(1→4)-β-D-oleandropyranosyl]-17β-marsdenin (P3), and 3-O-[6-deoxy-3-O-methyl-β-D-allopyranosyl-(1→4)-β-D-canaropyranosyl]-17β-marsdenin (P4) derived from *Gongronema latifolium* Benth. The results revealed that the SPPs source pregnane-rich chromatographic fractions and the SPPs (P1–P4) exhibited inhibitory potential against porcine pancreatic alpha-amylase *in vitro*. Compounds P1 and P2 with IC_50_ values 10.01 and 12.10 µM, respectively, showed greater inhibitory potential than the reference acarbose (IC_50_ = 13.47 µM). Molecular docking analysis suggests that the SPPs had a strong binding affinity to porcine pancreatic alpha-amylase (PPA), human pancreatic alpha-amylase (HPA), and human salivary alpha-amylase (HSA), interacting with the key active site residues through an array of hydrophobic interactions and hydrogen bonds. The strong interactions of the SPPs with Glu233 and Asp300 residues may disrupt their roles in the acid-base catalytic mechanism and proper orientation of the polymeric substrates, respectively. The interactions with human pancreatic amylase were maintained in a dynamic environment as indicated by the root mean square deviation, radius of gyration, surface accessible surface area, and number of hydrogen bonds computed from the trajectories obtained from a 100-ns molecular dynamics simulation. Key loop regions of HPA that contribute to substrate binding exhibited flexibility and interaction potential toward the compounds as indicated by the root mean square fluctuation. Furthermore, P1 significantly reduced blood glucose levels and area under the curve in albino rats which were orally challenged with starch. Therefore, *Gongronema latifolium* and its constituent SPPs may be exploited as inhibitors of pancreatic alpha-amylase as an oral policy for impeding postprandial blood glucose rise.

## Graphical Abstract

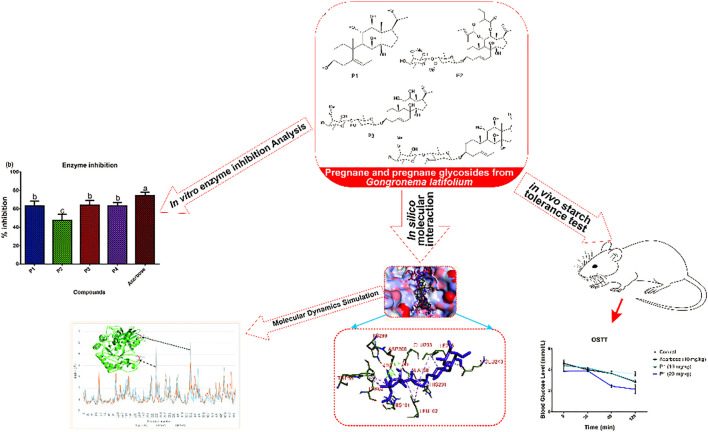

## Introduction

Diabetes mellitus (DM), a metabolic disease syndrome, is ranked among the persistent and most challenging public health burdens worldwide. It afflicts up to 463 million people globally and is projected to reach 700 million by 2045 (Cho et al., 2018; Kaur et al., 2021). In addition, DM has serious adverse effects on national economies (American Diabetes Association, 2014; Unuofin and Lebelo, 2020) as most countries in the world spend between 5% and 13% of their total budgetary allocation on diabetes (Zhang et al., 2010; Beseni et al., 2019). The global expenditure which was estimated at USD 376 billion in 2010 is expected to rise to about USD 490 billion by 2030 (Zhang et al., 2010). Untreated diabetes, which is characterized by prolonged postprandial and fasting hyperglycemia (Aryangat and Gerich, 2010; Riaz et al., 2020), may lead to dysfunction and failure of multiple organs (Mahomoodally et al., 2012; Beseni et al., 2019) and thereby results in several secondary complications such as neurodegenerative diseases, neuropathy, nephropathy, retinopathy, cardiovascular diseases among people living with DM (American Diabetes Association, 2014; Beseni et al., 2019).

A well-known therapeutic approach for the prevention and management of diabetes is decreasing diet-dependent blood glucose rise. This can be achieved by blunting dietary carbohydrate digestion in the gut through inhibition of digestive enzymes including alpha-amylase and alpha-glucosidase (Kim et al., 2005; Ogunyemi et al., 2022). The enzyme alpha-amylase (1,4-α-D-glucan glucanohydrolase, EC 3.2.1.1) is a key therapeutic target that has been exploited for developing several synthetic drugs such as acarbose, voglibose, and miglitol (Alqahtani et al., 2019). Several amylase inhibitors have also been identified from natural sources (Ogunyemi et al., 2020; Lankatillake et al., 2021; Ogunyemi et al., 2022). The alpha-amylase mainly occurring in the saliva and pancreas (Stiefel and Keller, 1973) helps to start the chemical process of carbohydrate breakdown by hydrolyzing the glycosidic bonds in starch and related substrate polysaccharides, transforming them into oligosaccharides and simple absorbable sugars. While the salivary amylase is produced by the salivary glands and initiates carbohydrate digestion in the mouth, a large amount of pancreatic amylase is secreted by the pancreas into the duodenum to continue the digestion of incoming starch. Human pancreatic α-amylase (HPA) is a 57 KDa protein comprising 512 amino acids in a single polypeptide chain with a well-defined active site region (Whitcomb and Lowe, 2007). Various synthetic inhibitors that interact with the active site and impair the catalytic function of this enzyme, as mentioned earlier, are known to be effective antidiabetics but are associated with several side effects such as flatulence, diarrhea, bloating, and abdominal discomfort (Kaur et al., 2021). This has stimulated researchers’ interest in developing safer alpha-amylase inhibitor molecules.

Many herbal extracts derived from herbs, spices, and medicinal plants with acknowledged and scientifically proven antidiabetic properties serve to complement current therapies in developing countries (Poovitha and Parani, 2016; Ogunyemi et al., 2022). The ethnopharmacological use of such herbs is increasingly gaining importance as medicines due to accumulating scientific evidence. They are also considered important vegetal resources for alpha-amylase inhibitors, preventive nutraceuticals, and antidiabetic drugs and may be exploited as an oral policy for impeding postprandial blood glucose rise in diabetes and metabolic syndrome (Dhital et al., 2013; Nyambe-Silavwe et al., 2015; Ogunyemi et al., 2022). Such antidiabetic plants and their activity against alpha-amylase were recently reviewed extensively by Kaur et al. (2021). *G. latifolium* Benth. is an antidiabetic plant that has been used in African traditional medicine against diabetic conditions (Al-Hindi et al., 2016; Ogunyemi et al., 2020). Several experimental pieces of evidence also support the ethnopharmacological use of this plant as the antidiabetic potentials of its bioactive extracts, and fractions have been documented *in vitro* and *in vivo* for over a decade (Akah et al., 2011; Chime et al., 2014; Al-Hindi et al., 2016; Ogunyemi et al., 2020). Such plants possessing antidiabetic activity mostly contain glycosides, pregnanes, alkaloids, terpenes, flavonoids, and carotenoids (Malviya et al., 2010; Ardalani and Hejazi Amiri, 2021). Major active constituents like pregnanes, pregnane glycosides, saponins, anthraquinones, alkaloids, β-sistosterol, sitostenone, lupenyl esters, glycosides, and essential oils have been isolated from different fractions of *G. latifolium* Benth. and may mediate biological activities of this food plant (Edet et al., 2009; Al-Hindi et al., 2016; Gyebi et al., 2018; Al-Hindi and Yusoff, 2019). On the other hand, several plant extracts containing pregnanes have been suggested and investigated as potential anti-obesity and antidiabetic drugs (Liu et al., 2013; Abdel-Sattar et al., 2018). For instance, the extracts and various fractions from the pregnane-rich *Caralluma* species, which are known to contain pregnane glycosides, were reported to show antihyperglycemic activity (Venkatesh et al., 2003; Habibuddin et al., 2008; Abdel-Sattar et al., 2011; Ashwini and Anitha, 2017). A recent report documented the inhibitory activity of five novel pregnane glycosides isolated from the ethanol extract of *Gymnema sylvestre* stem against α-amylase and α-glucosidase enzyme activities (Kiem et al., 2020). Therefore, plant pregnanes are dubbed as promising inhibitors of human pancreatic amylase, which may be exploited as preventive nutraceuticals for drug development. Herein, we used *in vitro*, *in silico*, and *in vivo* analyses to explore the alpha-amylase inhibitory potential of selected steroidal pregnane phytochemicals (SPPs) derived from *Gongronema latifolium*.

## Materials and methods

### Plant materials and phytochemicals

**FIGURE 1:**
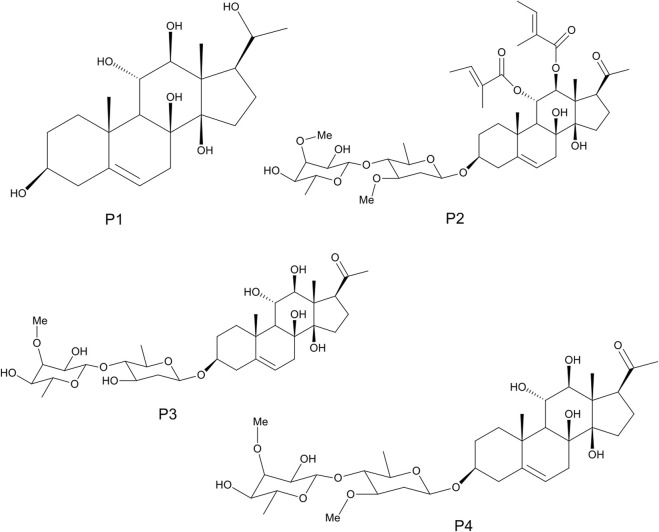
The 2D structures of marsectohexol (P1) and the pregnane glycosides, viz: iloneoside (P2), 3-O-[6-deoxy-3-O-methyl-β-D-allopyranosyl-(1→4)-β-D-oleandropyranosyl]-17β-marsdenin (P3), and 3-O-[6-deoxy-3-O-methyl-β-D-allopyranosyl-(1→4)-β-D-canaropyranosyl]-17β-marsdenin (P4) derived from *Gongronema latifolium* Benth.

The detailed methods of extraction, successive solvent-solvent partitioning, chromatographic fractionation, isolation, and characterization of bioactive compounds from *G. latifolium* have been reported in previous studies (Gyebi et al., 2018). Three bioactive pregnane-rich chromatographic fractions and the isolated steroidal pregnanes were selected for the current study. The isolated pregnane compounds comprise a pregnane marsectohexol (P1) and three pregnane glycosides, viz: iloneoside (P2), 3-O-[6-deoxy-3-O-methyl-β-D-allopyranosyl-(1→4)-β-D-oleandropyranosyl]-17β-marsdenin (P3), and 3-O-[6-deoxy-3-O-methyl-β-D-allopyranosyl-(1→4)-β-D-canaropyranosyl]-17β-marsdenin (P4) (Gyebi et al., 2018; Ogunyemi et al., 2020) as depicted in Figure 1.

### Enzyme inhibition assays

The alpha-amylase inhibitory activity of the pregnane-rich chromatographic fractions (F1–F3) and that of the isolated SPPs (P1–P4) alongside the reference acarbose were carried out as described by Carrasco et al. (2017) based on the dinitrosalicylic acid (DNSA) method (Miller, 1959). Varying concentrations of the pregnane-rich fractions (40–200 μg/ml) and those of the isolated compounds (10–50 μg/ml) were prepared in DMSO. Briefly, 100 μL of the test substance was reacted with 200 μl of alpha-amylase (alpha-amylase from porcine pancreas, EC 3.2.1.1, type VI, Sigma) and 100 μl of 2 mM of phosphate buffer (pH 6.9) of 0.02 M sodium phosphate (pH 6.9). Subsequent to incubation for 20 min, 100 μL of 1% starch substrate was added. Blank controls where the enzyme was replaced with the buffer were also prepared alongside. Following incubation of the reaction mixture for 5 min, 500 μl of dinitrosalicylic acid reagent was mixed with the control and the test. These were kept in a water bath at a boiling temperature for 5 min. The absorbance readings were taken at 540 nm, and the percentage inhibition was calculated as follows:

$$\mathrm{\% inhibition}=\;\frac{\left(\mathrm{Absorbanc}e_{\mathrm{control}}-\mathrm{Absorbanc}e_{\mathrm{test}}\right)}{\mathrm{Absorbanc}e_{\mathrm{control}}}\;\times 100,$$

where A_control_ = absorbance of the blank control (containing all reagents including 20% DMSO except the test solution) and A_test_ = absorbance of the test sample.

The half-maximal inhibitory concentration (IC50) values were determined by fitting inhibition parameters with standard log inhibitor vs. normalized response models using nonlinear regression as described by Buchwald (2020).

### Retrieval and preparation of proteins

The crystal structures of porcine pancreatic alpha-amylase (PDB ID: 1OSE), human pancreatic alpha-amylase (PDB ID: 4GQR), and human salivary alpha-amylase (PDB ID: 1SMD) were retrieved from the Protein Data Bank website: http://www.rcsb.org. The native ligands and molecules of water associated with the protein structures were removed, while missing hydrogen atoms were included using MGL-AutoDockTools (ADT, v1.5.6). The Kollman charges were included as the partial atomic charges (Morris et al., 2009). This procedure was applied to all proteins and then saved as the dockable protein databank extension pdbqt for docking simulations.

### Ligands preparation

The 3D structures of the SPPs, the reference inhibitor, and the co-crystallized compound were retrieved from the PubChem database (www.pubchem.ncbi.nlm.nih.gov) in structure file format (SDF). The chemical structures were then converted to mol2 format through Open Babel (O'Boyle et al., 2011). Then, polar hydrogen charges of the Gasteiger type were assigned to atoms, while the non-polar hydrogen molecules were merged with the carbon atoms. The ligands were then converted to the PDBQT format using AutoDock Tools for molecular docking simulation and molecular dynamics simulations.

### Molecular docking studies

Prior to docking analysis, the docking protocol was validated by redocking the native inhibitor into the active region of the enzyme. The binding pose with the minimal binding energy was superimposed on the retrieved co-crystallized inhibitor, after which the root mean square deviation (RMSD) was calculated using Discovery Studio. Then, the SPPs alongside reference inhibitors were docked into the active region of the enzymes using the AutoDock Vina docking tool in PyRx 0.8 (Trott and Olson, 2010). Blind docking calculations were also performed in order to screen the protein surface for possible binding spots other than the active site. The ligand molecules were imported, and energy was minimized using Open Babel (O'Boyle et al., 2011) in PyRx 0.8, applying the “universal force field” (UFF) as the energy minimization parameter and the conjugate gradient descent as the optimization algorithm. The parameters of the regions enclosing the active sites of the proteins as defined by the grid boxes are presented in Supplementary Table S1. The prepared enzyme structures were also imported, and the docking analysis was run with all other parameters kept as default. The docked complexes were visualized using the Discovery Studio Visualizer version 21 to detect and document the molecular interactions.

### Molecular dynamics simulations

Following molecular docking, apo-HPA and the complexes formed with the SPPs were subjected to a full atomistic 100-ns molecular dynamic (MD) simulation in GROMACS 2019.2 using GROMOS96 43a1 forcefield on the WebGRO (Bekker et al., 1993; Oostenbrink et al., 2004; Abraham et al., 2015). The required topology files of the ligand molecules were generated using the PRODRG web server (http://davapc1.bioch.dundee.ac.uk/cgi-bin/prodrg) (Schüttelkopf and Van Aalten, 2004). The solvation of the apoenzyme and ligand–enzyme complex systems within a cubic box of the transferable intermolecular potential was performed with a four-point (TIP4P) water model, employing the periodic boundary conditions at a physiological concentration of 0.154 M set by neutralized NaCl ions. The minimization of the biomolecular systems was performed for 10,000 steps using the steepest descent algorithm in a constant number of atoms, volume, and temperature (NVT) ensemble for 0.3 ns followed by 0.3 ns of equilibration in constant atom number, constant pressure, and constant temperature (NPT). The temperature was maintained using 310 K using velocity rescale, while pressure was set to 1 atm using Parrinello–Rahmanbarostat. Leap-frog integrator was used with a time step of two femtoseconds. For the 100 ns production run performed for each system, a 0.1-ns snapshot was saved with a total of 1,000 frames from each system. The thermodynamic parameters such as RMSD, RMSF, SASA, RoG, and the number of H-bond were computed from the trajectory files using VMD TK console scripts (Humphrey et al., 1996).

### Oral starch tolerance test

The animal study was reviewed and approved by the University Ethical Review Committee of the University of Ilorin (Protocol Identification Code: UERC/LSC/183). An oral starch tolerance test was carried out as described in a previous study (Poovitha and Parani, 2016). Sixteen fasted albino rats were divided into four groups of four each, and orally treated with DMSO (negative control), 10 mg/kg acarbose (positive control), 10 mg/kg marsectohexol, and 20 mg/kg marsectohexol. After 10 min, the blood glucose level was estimated (0 min), and the rats were orally administered with 3.0 g/kg starch. Then, the blood glucose level was estimated at 30, 60, and 120 min after the administration using a glucometer. Peak blood glucose (PBG) was determined by observing the blood glucose level during the previously mentioned time intervals, and the area under the curve (AUC) was calculated.

### Statistical analysis

Data were processed using Microsoft Excel 2010, Graphpad software 6.0, and SAS 9.12 statistical software. One-way analysis of variance (ANOVA) and Duncan multiple range were used to assess the possible differences among the means. The results were expressed as the mean ± SEM and *p* < 0.05 as the level of statistical significance.

## Results

### *In vitro* analysis of amylase inhibition by pregnane-rich fractions and steroidal pregnane phytochemicals

**FIGURE 2:**
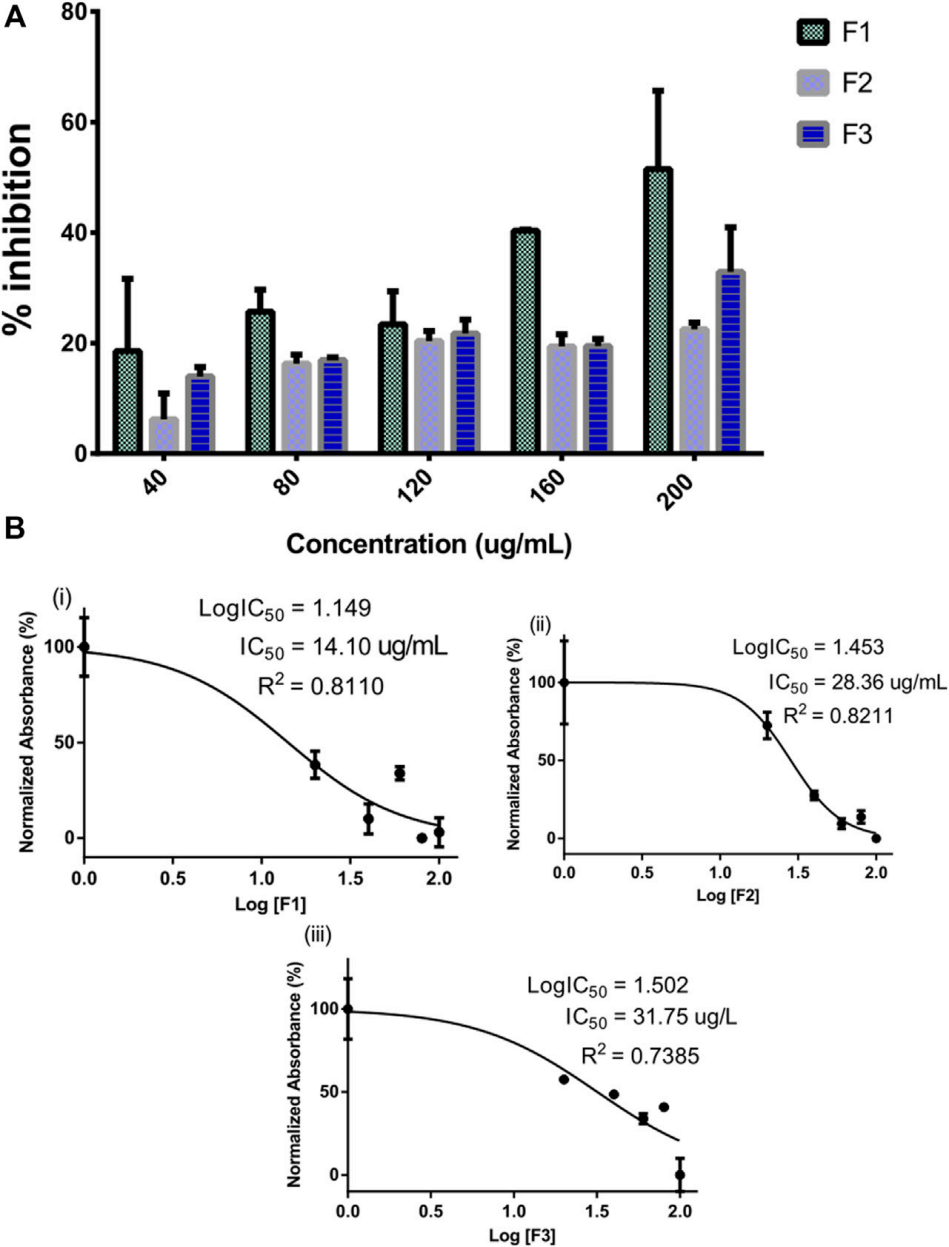
Inhibitory activity of bioactive fractions of *Gongronema latifolium* Benth. against alpha-amylase. Percentage inhibitory activity of the selected pregnane-rich chromatographic sub-fractions on porcine alpha-amylase **(A)**. Half-maximal inhibitory concentrations (IC50) of the pregnane-rich fractions **(B)**.

The inhibitory potential of selected pregnane-rich chromatographic sub-fractions derived from *G. latifolium* Benth. is depicted in Figure 2.

The fraction F1 exhibited the strongest alpha-amylase inhibition in a concentration-dependent manner (Figure 2A). This fraction possessed an IC_50_ value of 14.10 μg/ml (Figure 2B). Fractions F2 and F3 also possessed considerable inhibitory potential against the target enzyme.

**FIGURE 3:**
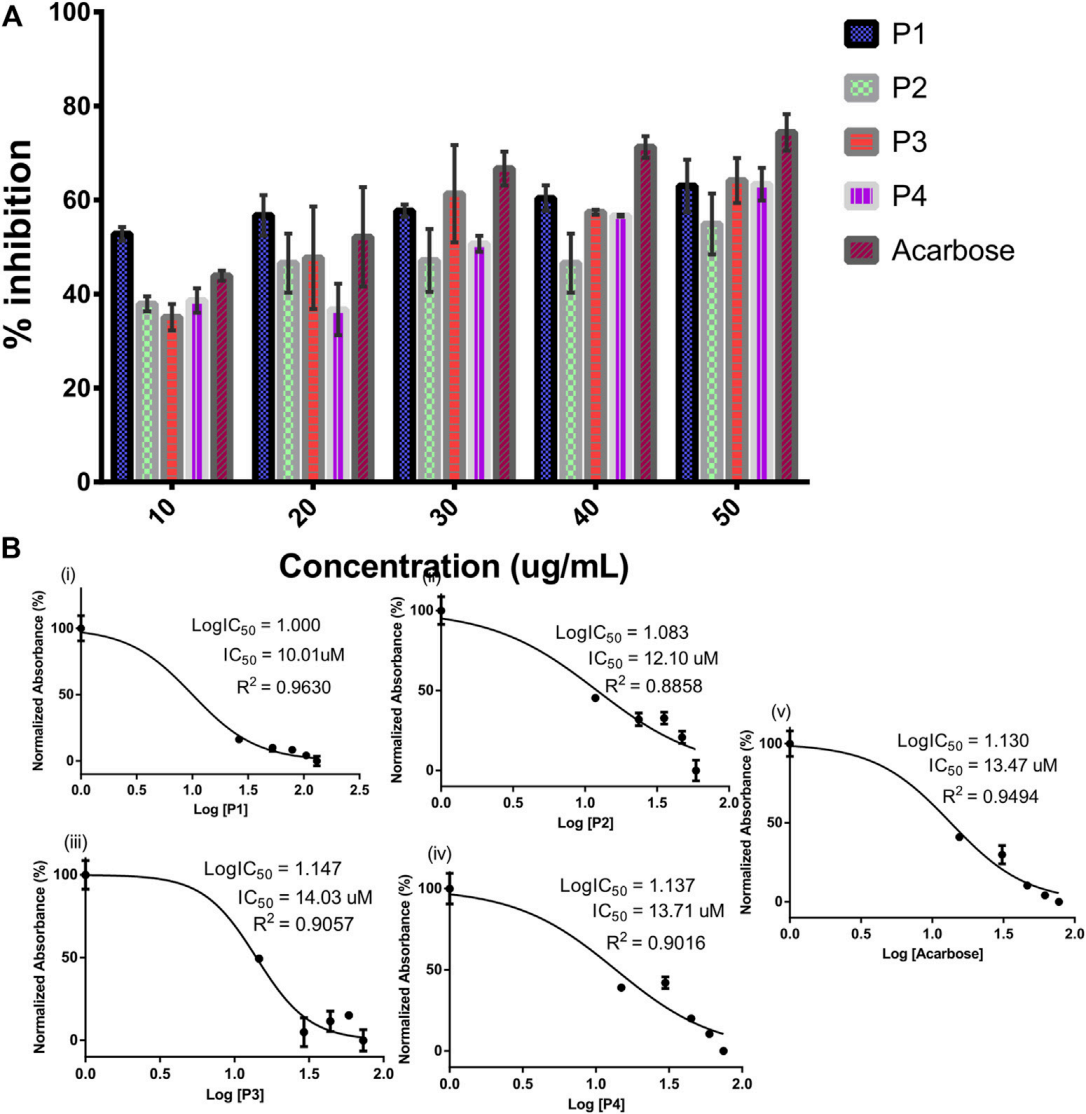
Inhibitory activity of steroidal pregnane compounds (P1–P4) and acarbose against alpha-amylase. Percentage inhibitory activity **(A)** and half-maximal inhibitory concentrations (IC50) **(B)**.

The SPPs derived from *G. latifolium* Benth. in comparison with the reference acarbose showed inhibitory potential against porcine alpha-amylase as depicted in Figure 3.

Although the reference acarbose exhibited the greatest inhibition effects, compounds P1 and P2 with IC_50_ values of 10.01 and 12.10 µM, respectively, showed greater inhibitory potential than the reference acarbose (IC_50_ = 13.47 µM).

### Molecular docking simulation

**FIGURE 4:**
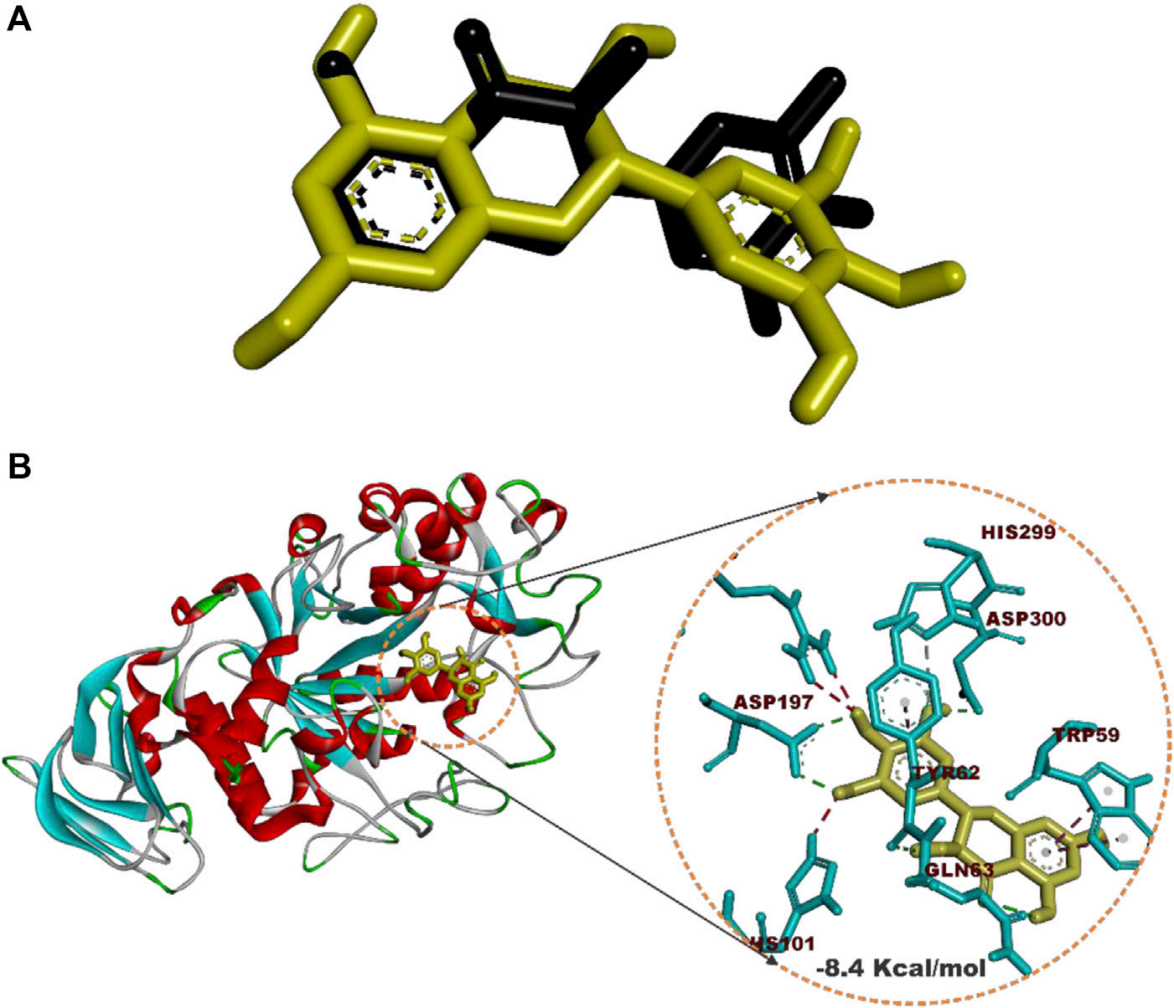
Redocked native compound: **(A)** Superimposition of selected docked conformer of co-crystallized myricetin on the retracted co-crystallized structure. **(B)** interacted amino acid residues with myricetin.

Prior to molecular docking simulation, which was based on the scoring function in AutoDock Vina, the docking protocol was validated by retracting myricetin, the co-crystallized compound with the human pancreatic alpha-amylase protein structure (PDB ID: 4GQR). The compound was subsequently redocked into the same domain of the enzyme structure. The result revealed that all the docking conformations of myricetin structure were located within the active site region of the enzyme and interacted with the catalytic triad and other critical residues that define the active site of the enzyme as shown in Figure 4.

Estimation of the root mean square deviation (RMSD) between the lowest binding pose and the initial crystal structure gave 1.97 Å (less than 2.00 Å), while the best-docked conformation of myricetin with HPA had a docking score of −8.4 Kcal/mol and interacted with HPA through hydrogen bonds with Asp197, Asp300, Tyr62, Gln63, and His299 and hydrophobic interactions with Trp59 and Tyr62.

Subsequent to docking validation, an active-site directed docking of the compounds against the structures of porcine pancreatic amylase, human pancreatic amylase, and human salivary amylase was conducted. Blind docking, which helps to screen the whole surface of the proteins, was also conducted to assess possible binding interactions to other sites such as regulatory sites. The docking scores obtained from this analysis are shown in Table 1.

**TABLE 1:** Docking scores of the targeted and blind docking simulations of pregnanes with porcine alpha-amylase and human amylase.

Compounds	PPA		HPA		HSA	
	Targeted score (Kcal/mol)	Blind score (Kcal/mol)	Targeted score (Kcal/mol)	Blind score (Kcal/mol)	Targeted score (Kcal/mol)	Blind score (Kcal/mol)
Acarbose (E = 852.22)	−6.1	−8.2	−6.5	−7.1	−6.2	−7.2
P1 (E = 1048.30)	−8.4	−8.5	−8.4	−8.4	−7.7	−7.7
P2 (E = 1688.06)	−0.8	−8.5	−7.7	−9.2	−7.9	−8.0
P3 (E = 1455.05)	−5.4	−9.3	−8.1	−9.0	−9.0	−9.3
P4 (E = 1489.24)	−5.9	−9.4	−8.5	−8.4	−8.9	−8.9

[E = Energy of minimization in universal force field (UFF)**]**.

Molecular docking of the SPPs and acarbose with the active site of PPA revealed that the P1 had the lowest docking score (−8.4 Kcal/mol) as compared to the pregnane glycosides (P2–P4) and the reference acarbose (−6.1 Kcal/mol). The blind docking revealed comparable binding energy of the pregnanes to PPA. All the SPPs had lower docking scores than the reference acarbose with respect to both targeted docking and blind docking to HPA. Similar results were obtained in the case of HSA.

### Molecular interactions of the pregnane compounds with alpha-amylase

The SPPs are C21 steroid derivatives with carbons present at positions 1 through 21 (Figure 1). Several interactions were formed between these structures and the active site residues of the enzyme proteins are shown in Table 2.

**TABLE 2:** Molecular interactions of porcine pancreatic alpha-amylase, human pancreatic alpha-amylase, and human salivary alpha-amylase with acarbose and the pregnane compounds (P1–P4) derived from *G. latifolium*.

	Enzymes	Active site docking				Blind docking			
Compounds		Hydrogen bond interactions		Hydrophobic interaction		Hydrogen bond interaction		Hydrophobic interaction	
		No	Residues	No	Residues	No	Residues	No	Residues
Acarbose	PPA	4	Asp300 (3) and Gly306	3	Lys200, Ile235, and Tyr151	3	Arg252, Lys278, and Glu404	1	Pro332
P1		3	His 299 and **Asp 300** (2)	10	Leu162 (2), Ala198, Ile235, Leu165, Trp58, Tyr62 (3), and His201	6	His 299, Gly306, **Asp300(2)**, and **Glu233 (2)**	8	Leu162 (2), Ala198, Leu165, Trp58, Tyr62 (2), and His201
P2		1	His299	7	Val163(2), Leu162 (2), Lys200, His201, and His299	6	Arg252, Trp280, Gly334, His331, Asn279, and Trp280	4	Pro332 (2), Pro405, and Phe335
P3		1	His299	4	Leu162, Ile235, and Tyr62	8	Arg252, Ser289, Pro332, Gly334, His331 (2), Asn279, and Trp280	3	Pro4 and Phe335 (2)
P4		5	**Asp300** (3) and Glu240 (2)	9	Leu162 (2), Ala198, Ile235, Ala307, Trp58, Tyr62 (2), and His201	5	Lys200, Glu240, **Glu233 (2)**, and **Asp300**	8	Leu162 (2), Ala198, Ile235, Ala307, Leu237, Tyr62, and His201
Acarbose	HPA	8	**Lys200, His305 (2), Asp300 (3),** and **Glu233 (2)**	2	**Trp58** and **Trp59**	6	Glu233 (3), Asp300, His305, and Gly306	2	Trp58 and Trp59
P1		3	Glu233 (2) and Asp300	8	**Leu162 (2), Leu165 (2), Ala198, Ile235, Leu162,** and **His201**	4	**Asp197, Glu233,** Ala198, and Gln63	10	Leu162 (3), Leu165 (2), Ala198, Ile235, Trp59, His201, and Gln63
P2		3	Asp197, **Thr163,** and **Tyr151**	10	**Tyr151 (3), Ile235 (2), Trp58 (2), Trp59, His299,** and **His305**	4	**Asp300**, Thr163, and Glu240	9	Tyr151, Trp59 (4), Leu162, Tyr62, His201, and His305
P3		3	Thr163 and **Asp300 (**2)	11	Leu162, (2), Ile235 (2), Trp58, Trp59 (2), Tyr62, Tyr151, and His201	3	Thr163, Glu240, and Gly239	15	Leu162 (2), Ala198, Ile235 (2), Trp58, Ala307, Leu237 (2), Tyr62 (2), and His201 (2)
P4		6	**Glu233,** Glu240 (2), **Asp300** His305, and Gly306	10	Leu162 (2), Ala198, Ile235, Trp58, Tyr62 (2), Tyr151 (2), and His201	5	Thr163, **Asp300** (2), His305, and Gly306	6	Leu162, Ile235, Leu162, Leu237, Tyr62, and Tyr151
Acarbose	HSA	3	**Asp300,** Gly306, and Lys200	3	Trp58, Trp59, and His305	5	**Asp300** (2), Arg195, and Lys352 (2)	1	Trp59
P1		2	**Glu233** and **Asp300**	7	Leu162 (2), Ala198, Leu165, Tyr62, and His201 (2)	3	**Asp300** (3)	10	Leu162 (2), Leu165, Ala198, Trp58, Tyr62 (2), His101, and His201
P2		3	**Glu233** and **Asp300** (2)	9	Trp59 (2), Leu162 (2), Ala198, Ala307, Tyr62, and His201 (2)	5	**Asp300,** Ser163, His305, and Leu237	7	Tyr151, Trp59 (3), Leu162 (2), and His305
P3		3	**Asp300** (2) and Glu240	12	Leu162 (2), Ala198, Ala307, Ile235, Leu237, Trp58, Tyr62 (2), His101, and His201 (2)	5	**Glu233,** Asp300 (2), Glu240, and Lys200	11	Leu162 (2), Ala198, Ala307, Ile235, Trp58, Tyr62 (2), His101, and His201 (2)
P4		4	**Asp300** (3) and Glu240	10	Leu162 (2), Ala198, Ile235 (2), Trp68, Tyr62 (2), His101, and His201	3	**Asp300** (2) and Glu240	10	Leu162 (2), Ala198, Ile235 (2), Trp58, Tyr62 (2), His101, and His 201

NB: P1, marsectohexol; P2, 3-*O*-[6-deoxy-3-*O*-methyl-β-D-allopyranosyl-(1→14)-β-D-oleandropyranosyl]-11,12-di-*O*-tigloyl-17β-marsdenin; P3, 3-O-[6-deoxy-3-O-methyl-β-D-allopyranosyl-(1→4)-β-D-oleandropyranosyl]-17β-marsdenin; P4, 3-O-[6-deoxy-3-O-methyl-β-D-allopyranosyl-(1→4)-β-D-canaropyranosyl]-17β-marsdenin. Amino acid residues in bold font are members of the catalytic triad. Figures in parenthesis indicate multiple bonds exhibited by the residues. Amino acids in bold fonts are the catalytic residues.

**FIGURE 5:**
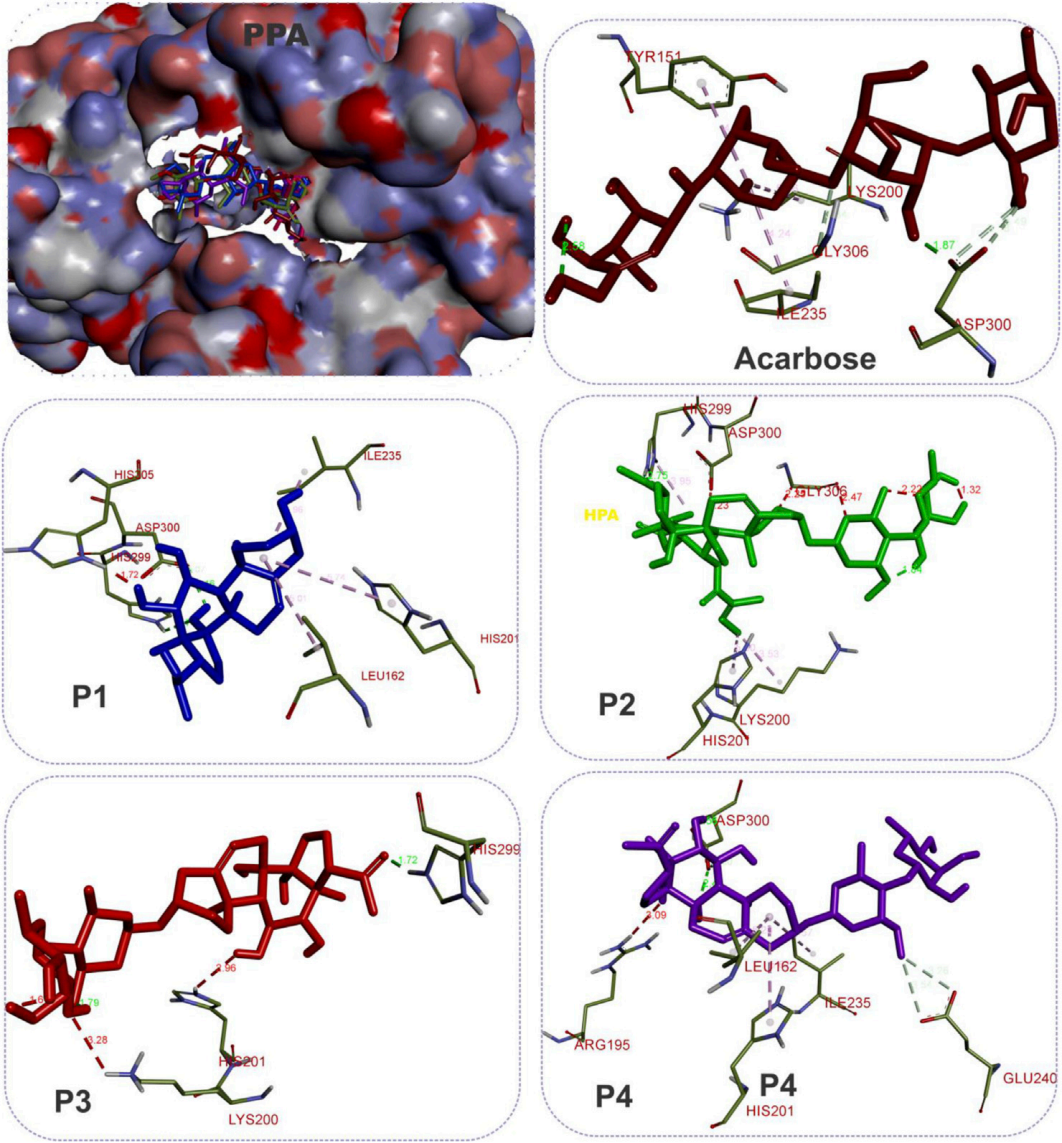
The 3D depiction of the interactions of acarbose and the isolated steroidal pregnanes with amino acid residues in the active site of porcine pancreatic alpha-amylase (PPA). Ligands are depicted in stick representations in colors red: acarbose (reference inhibitor); blue: P1; green: P2; pink: P3; purple: P4. Interaction types (dotted lines) and their bond lengths are depicted in green (H-bonds); light purple: hydrophobic interactions (pi-alkyl, alkyl, and pi-stacking); purple: pi-pi T-shaped interactions; yellow: pi-sulfur interactions and pi-stacking interactions. Amino acid residues are in a three-letter representation*.*

**FIGURE 6:**
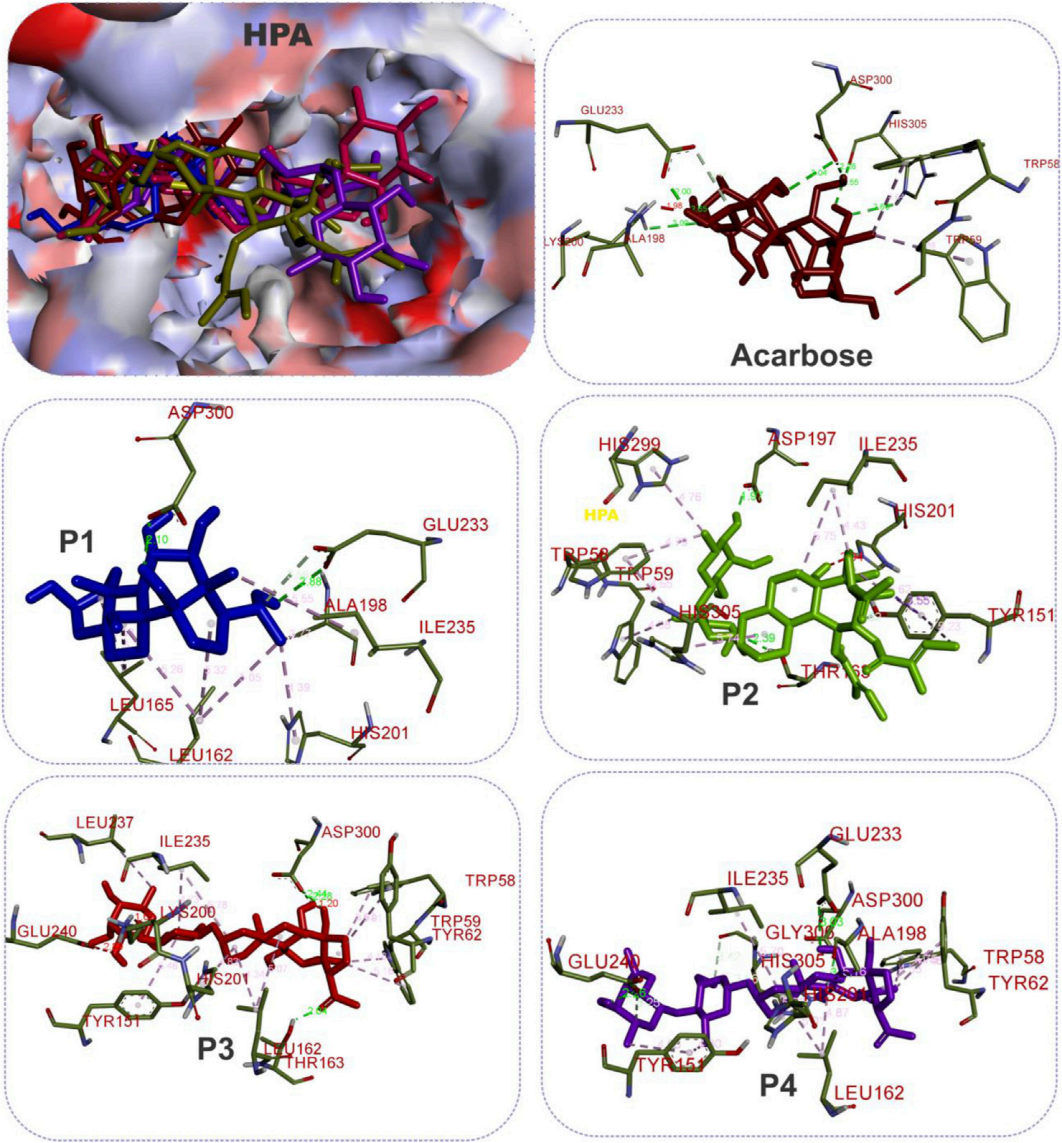
The 3D depiction of the interactions of acarbose and the isolated steroidal pregnanes with amino acid residues in the active site of human pancreatic alpha-amylase (HPA). Ligands are depicted in stick representations in colors red: acarbose (reference inhibitor); blue: P1; green: P2; pink: P3; purple: P4. Interaction types (dotted lines) and their bond lengths are depicted in green (H-bonds); light purple: hydrophobic interactions (pi-alkyl, alkyl, and pi-stacking); purple: pi-pi T-shaped interactions; yellow: pi-sulfur interactions and pi-stacking interactions. Amino acid residues are in a three-letter representation.

**FIGURE 7:**
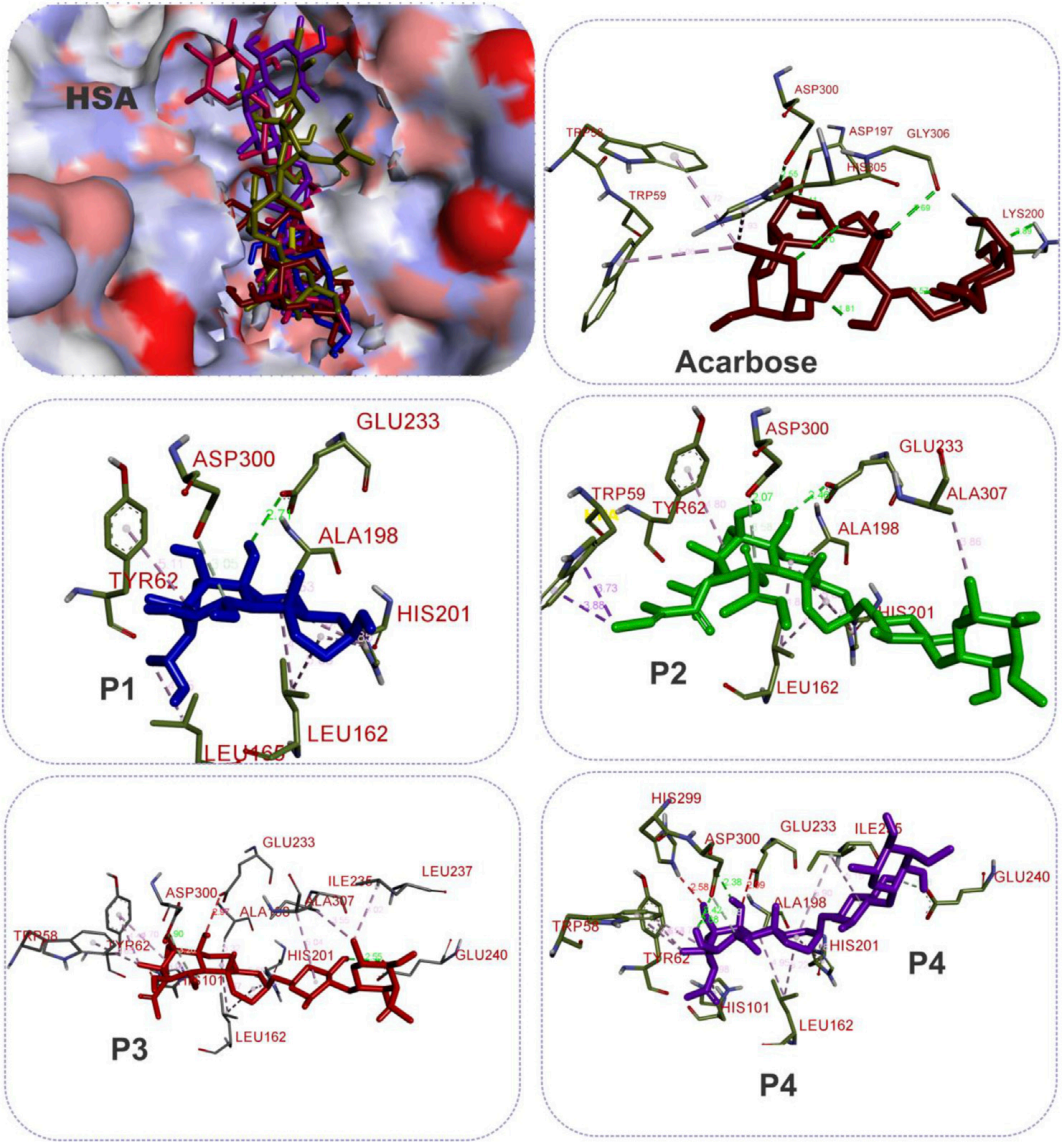
The 3D depiction of the interactions of acarbose and the isolated steroidal pregnanes with amino acid residues in the active site of human salivary alpha-amylase (HSA). Ligands are depicted in stick representations in colors red: acarbose (reference inhibitor); blue: P1; green: P2; pink: P3; purple: P4. Interaction types (dotted lines) and their bond lengths are depicted in green (H-bonds); light purple: hydrophobic interactions (pi-Alkyl, alkyl, and pi-stacking); purple: pi-pi T-shaped interactions; yellow: pi-sulfur interactions and pi-stacking interactions. Amino acid residues are in a three-letter representation.

The results showed that while the interactions of acarbose with the active site were dominated by hydrogen bonds, the interactions of the SPPs were dominated by hydrophobic interactions. The amino acid interactions reported in Table 2 are further illustrated graphically in surface view and three-dimensional structure to show the bond sub-types and bond distances as depicted in Figures 5–7. Figure 5 shows that the SPPs fit into the binding pocket of the porcine alpha-amylase and interacted with critical amino acids that define the active site of the enzyme. In a similar manner, the compounds interacted with the active site of human enzymes as depicted in Figures 6, 7.

### Molecular dynamics simulation

**FIGURE 8:**
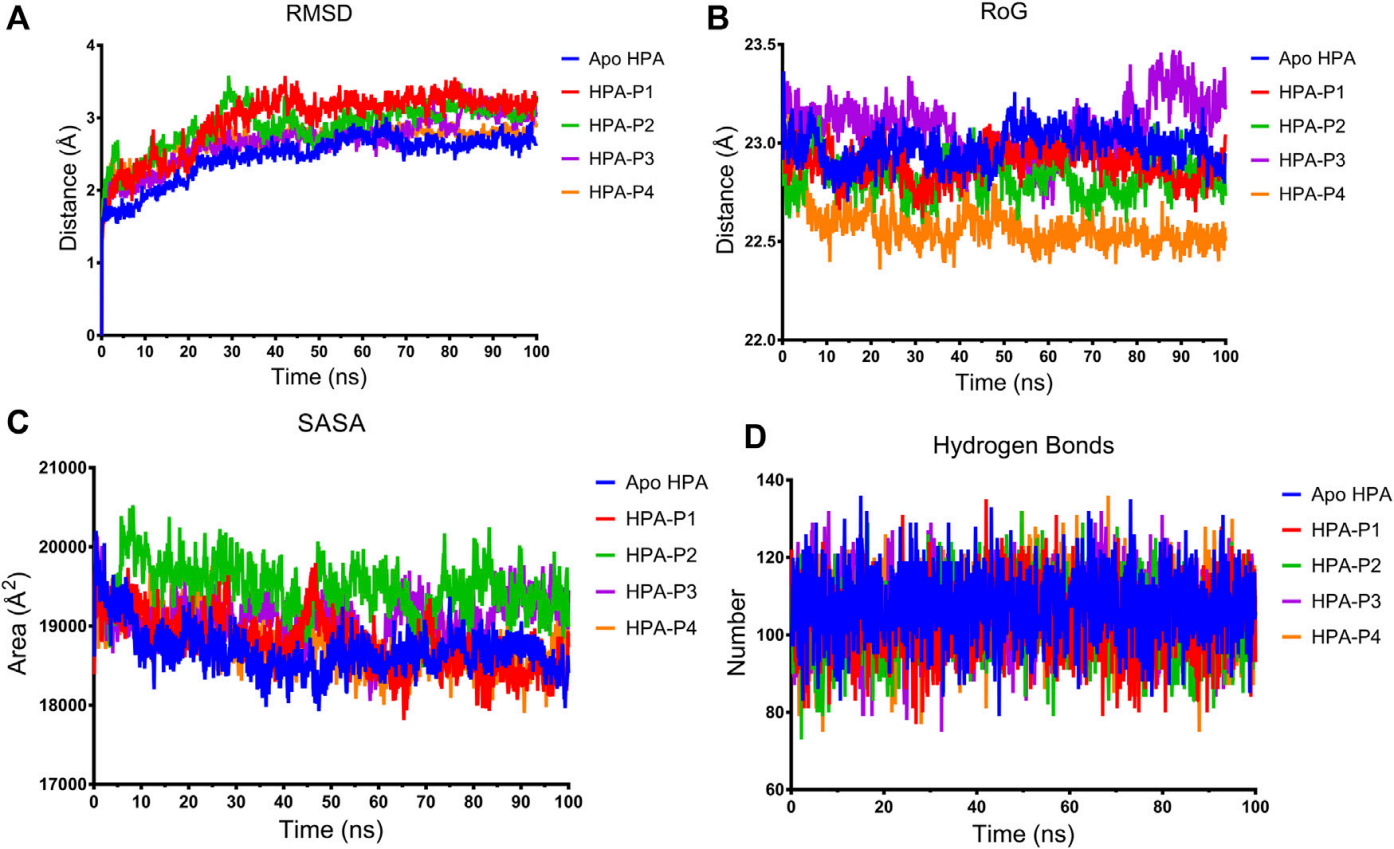
Structural stability of apo human pancreatic amylase and in complex with the isolated steroidal pregnanes. **(A)** Backbone root mean square deviation (RMSD) plots. **(B)** Radius of gyration (RoG) plots. **(C)** Surface accessible surface area (SASA) plots. **(D)** Change in the number of hydrogen bonds.

**FIGURE 9:**
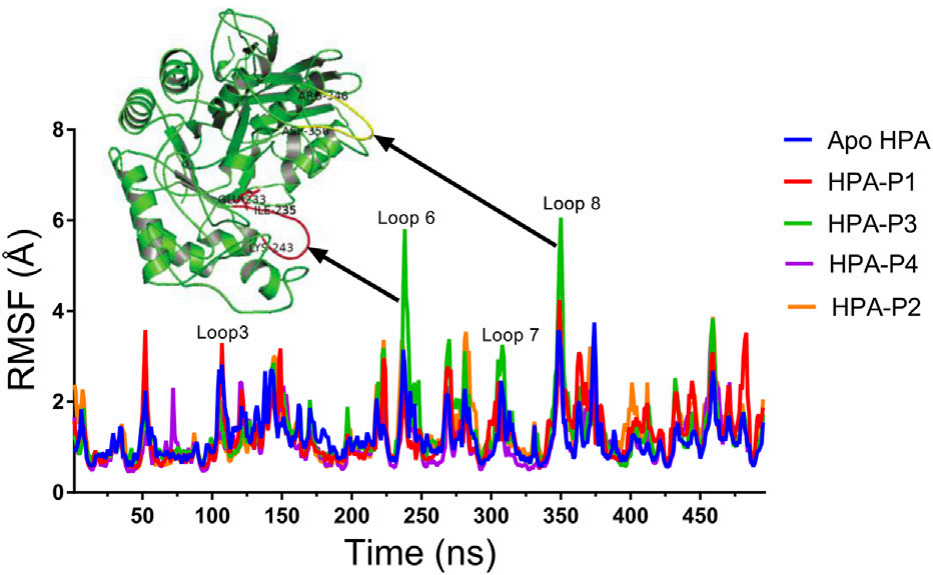
Per residue root mean square fluctuations (RMSF) plots of molecular dynamics (MD) simulation of human pancreatic amylase (HPA) and in complex with the isolated steroidal pregnanes.

In this study, a 100-ns atomistic MDS was performed to compare the stability and structural conformation of the ligand–enzyme complexes with unbound enzyme (apoenzyme) in a dynamic environment as indicated by the thermodynamic parameters computed and plotted from the MDS trajectory files (Figures 8, 9).

The root mean square deviation (RMSD) of the backbone C-α atoms of all the HPA systems showed a stable trend after equilibration as depicted in Figure 8A. The RMSD of the backbone C-α atoms of the apo-HPA showed an increasing trend in the first 21.9 ns before stabilizing at around an average value of 2.61 Å. The HPA-P1 system showed the rising value of RMSD until 24.5 ns at which stability around 3.18 Å was attained. The HPA-P2 system showed stability beyond 28.8 ns, averaging at 3.02 Å. Stability was attained for HPA-P3 at 25 ns with an average value of 2.83 Å. The HPA-P4 system attained stability around 16.6 ns beyond which RMSD averaged at 2.76 Å. Upon equilibration and stabilization, the RMSD values were maintained within 2 Å. The radius of gyration (RoG) plot further showed stability of the ligand–HPA complexes (Figure 8B). The RoG value of the apo-enzyme system was maintained around 22.98 Å, while those of the HPA-P1, HPA-P2, HPA-P3, and HPA-P4 were maintained around 22.89, 22.82, 23.08, and 22.57 Å, respectively. Furthermore, the surface accessible surface area (SASA) plot in Figure 8C indicates the level of the solvent accessibility surface of the protein systems. The apo-enzyme system exhibited an average SASA value of around 18,699.95, while HPA-P1, HPA-P2, HPA-P3, and HPA-P4 showed 18,781.49, 19,491.29, 19,120.87, and 18,766.17 Å^2^, respectively. A more detailed stability analysis through computation of the number of hydrogen bonds existing in the biomolecular systems was performed, and the plot is shown in Figure 8D. The number of H-bonds shows a stable trend for all the biomolecular systems. The apo-HPA, HPA-P1, HPA-P2, HPA-P3, and HPA-P4 featured 108, 104, 103, 106, and 106 hydrogen bonds, respectively.

The RMSF in Angstrom (Å) was plotted for the apo-HPA and in complex with the isolated steroidal pregnanes (Figure 9).

Compared to the non-complexed HPA, increased disorder was observed in specific loop regions of the proteins. Some of the amino acid residues that define the active site of HPA showed significant fluctuations (greater than 2 Å). In addition to the active site residues, other residues also show several significant peaks of increased RMSF. Specific regions of the RMSF plots that showed the highest fluctuations are the Phe348–Asn352 region (yellow cartoon) as well as the Gly238–Glu240 region (red cartoon). These regions form loops in the vicinity of the active site of the enzyme. These loops showed the highest fluctuations upon interactions with the compound P3.

### Oral starch tolerance test

**FIGURE 10:**
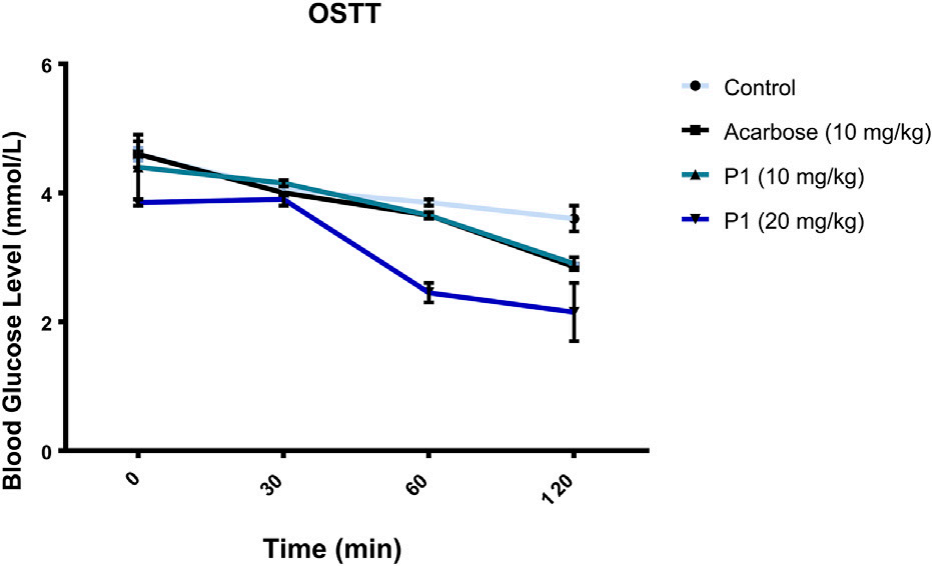
Effects of P1 (marsectohexol) from *Gongronema latifolium* and acarbose on the blood glucose level after the administration of 3 mg/kg starch to albino rats. Values are the mean ± SEM (*n* = 4), at *p* < 0.05.

The orally administered marsectohexol alongside acarbose showed a significant decrease in the blood glucose level as depicted in Figure 10, as well as the peak blood glucose and area under curve (Table 3), further indicating the inhibitory role of this compound against alpha-amylase.

**TABLE 3:** Effect of acarbose and the marsectohexol on blood glucose level in albino rats.

Group	PBG (mmol/L)	% reduction of PBG	AUC (mmol/L)	% reduction of AUC
Control	3.60 ± 0.20^a^		943.5 ± 10.5^a^	
Acarbose (10 mg/kg)	2.85 ± 0.05^ab^	20.83	877.5 ± 4.50^a^	7.00
Marsectohexol (10 mg/kg)	2.90 ± 0.10^ab^	19.44	883.5 ± 19.50^a^	6.36
Marsectohexol (20 mg/kg)	2.15 ± 0.45^b^	28.18	699.0 ± 48.0^b^	25.91

PBG, peak blood glucose; AUC, area under the curve. Values are the mean ± SEM (*n* = 4), at *p* < 0.05 vs. control.

## Discussion

Alpha-amylase is widely exploited as a drug target for preventing postprandial hyperglycemia in diabetes and other metabolic diseases (Borah et al., 2019; Ogunyemi et al., 2022). Pregnane-rich plant extracts are known to possess strong alpha-amylase inhibitory potential and thereby serve as important bioresources for preventive nutraceuticals and potent antidiabetic agents as discussed earlier. Herein, the alpha-amylase inhibitory action of selected pregnane-rich chromatographic fractions and steroidal pregnane phytochemicals (SPPs) derived from *G. latifolium* Benth. was reported. The observed inhibitory activity of the pregnane-rich plant extracts against the porcine pancreatic amylase *in vitro* (Figure 2) and that of the isolated SPPs (Figure 3) was expected as the fraction F1 (previously combined Fr.5 and Fr.6) is the source chromatographic fraction of compounds P1 and P2, while the F3 (derived from further fractionation of Fr.8) afforded P3 and P4 as reported earlier (Gyebi et al., 2018; Ogunyemi et al., 2020). Depending on the experimental conditions, several studies have reported the range of alpha-amylase and alpha-glucosidase inhibitory activity of acarbose as 55%–82% (Mohamed et al., 2012; Perez-Gutierrez and Damian-Guzman, 2012; Olubomehin et al., 2013). Under the same experimental conditions, the SPPs showed high inhibitory activity against α-amylase but less than reference acarbose. Our study is in tandem with a previous *in vitro* study that demonstrated the inhibition of carbohydrate hydrolyzing enzymes by eight pregnanes derived from the methanolic extracts of the fruit pericarp of *Gymnema griffithii*.

(Srisurichan et al., 2014). Also, russelioside B, a pregnane glycoside structure isolated from *Caralluma quadrangula*, was reported to exhibit antihyperglycemic activity in streptozotocin-induced diabetes albino rats through regulation of important carbohydrate metabolizing enzymes (Abdel-sattar et al., 2016). In a previous study that employed porcine pancreatic amylase and human pancreatic amylase, bisdemethoxycurcumin from *C. longa* inhibited these enzymes in a similar manner (Ponnusamy et al., 2012). We reported that all the SPPs showed good IC_50_ values against porcine alpha-amylase *in vitro*, some of which were on par with the reference acarbose. The compound P1 (Figure 3) exhibited the lowest IC_50_ value (10.01 µM) as compared to reference acarbose (IC_50_ = 13.47 µM) and other SPPs. Compound P2 with an IC_50_ value of 12.10 µM also showed stronger inhibitory potential than the reference acarbose. Structurally, compound P1 possesses a C21 steroidal framework with carbons present at positions 1 through 21 (Figure 1). It is unglycosylated unlike the compounds P2–P4 with a sugar moiety attached to the alcohol group at C-3 of the steroidal core. P2 differs from the other glycosylated steroidal pregnanes (P3 and P4) owing to the presence of a tigloyl component attached at C-11 and C-12.

Given the bulky structures, which may confer the ability to bind to multifarious biological targets (Sales et al., 2012; Saini et al., 2016; Saini et al., 2017) and the structural differences of the SPPs, *in silico* study was performed to assess the interactions of the compounds with alpha-amylase structures with well-defined active site regions. The polypeptide chain of an alpha-amylase is known to fold into three domains, viz: domains A, B, and C, starting at the amino terminal (Brayer et al., 1995). The enzyme active site occurs as a pocket found in the (β/α)_8_ barrel motif in domain A of the polypeptide chain while the domain B comprises an important loop that occurs between the third β-strand and the third helix of the (β/α)_8_ barrel (Buisson et al., 1987; Nahoum et al., 2000). The active site of this enzyme comprises five sub-sites which bind different glucose residues in the substrate (Seigner et al., 1987). Prior to the docking analyses of the SPPs with PPA, HPA, and HSA, the docking protocol was validated by redocking the native myricetin with HPA. The low RMSD value observed between the lowest binding pose and the experimental crystal structure, which is significantly below 2.5 Å, is an indication of the accuracy of the molecular docking protocol (Elekofehinti et al., 2021; Iwaloye et al., 2021). In addition to the low RMSD, the low binding score, the favorable docked conformations, and interactions with the active site amino acid residues observed further showed that myricetin successfully mimicked the experimental structures determined by X-ray crystallography. Thus, the docking protocol using AutoDock Vina software is reliable. Application of the docking protocol to the pregnane compounds revealed that the best-docked conformations of the pregnane compounds had good docking scores, which were comparable to or lower than those of reference acarbose. This suggests strong binding interactions of the SPPs with the PPA, HPA, and HSA. A closer inspection of the binding interactions of acarbose and the SPPs with the target enzymes revealed desirable interactions with the active site region of the enzyme, while the interactions of acarbose with the active sites were dominated by hydrogen bonds and interactions of the SPPs were dominated by hydrophobic interactions. This might be due to the steroid core structure which typically comprises carbon atoms bonded in four “fused” rings: three six-member cyclohexane rings (rings A, B, and C) and one five-member cyclopentane ring (the D ring). The X-ray crystal structure and biochemical studies of amylase have revealed the catalytic triad comprising Asp197, Glu233, and Asp300 (Nahoum et al., 2000; Rydberg et al., 2002; Williams et al., 2012). Asp197 is known to intervene in the nucleophilic reaction (Rydberg et al., 2002), while residue Glu233 mediates the acid-base catalysis, and Asp300 involves the optimized orientation of the substrate in the binding site. The active site-directed docking and blind docking simulations performed in this study show several overlaps of amino acid interactions. This might be due to binding at both the active site and other pockets in close proximity to the binding site of the enzymes. Dandekar et al. (2021) recently identified eight consensus surface pockets (site 1–site 8) on the HPA structure, with site 1 having a partial overlap with the known active site of HPA with common residues Asp197, Glu233, and Asp300, while the pocket site 2–site 8 are distinctly away from the active site. The major overlaps observed in this study involve Glu233 and Asp300. Previous reports have delineated several inhibitory mechanisms known to underpin the actions of known alpha-amylase inhibitors. These include the potency to construct hydrogen bonds and other interactions with the catalytic triad (Asp197, Glu233, and Asp300) and other catalytic residues, interactions at sites other than the active site, or covalent bond with enzymes through an epoxy or aziridine group (Moorthy et al., 2012; Rouzbehan et al., 2017). The interaction analysis of the SPPs and reference acarbose showed that they have a close binding pattern as the native myricetin and interacted with the catalytic residues through hydrogen bonds and hydrophobic interactions. The acarbose structure conducted both hydrogen bonds and hydrophobic interactions with the active site residues of the target enzyme as reported in previous studies (Singla et al., 2016; Etsassala et al., 2020; Ogunyemi et al., 2020). The hydroxyl groups on C-20 and C-14 of the pregnane structure of the P1 donated hydrogen to the side chains of Glu233 and Asp300 of HPA, respectively, with bond distances less than 2.9 Å to form conventional hydrogen bonds. The C-20 on the ring D of the steroid structure of this compound made an additional carbon-hydrogen contact (bond distance, 3.56 Å) with the side chain of Glu233. Strong interactions with Glu233 may have negative impacts on its critical role in the acid-base catalytic mechanism of the enzyme. Also, interactions with Asp300 may hinder the proper orientation of the polymeric substrates of the enzyme as illustrated in previous studies (Nahoum et al., 2000; Rydberg et al., 2002; Williams et al., 2012). In addition to these strong interactions, the P1 structure also made alkyl hydrophobic interactions with several amino acids such as Ala198, Ile235, Leu165, and Leu162. The C21 made pi-alkyl interaction with His201. A similar study by Williams et al. (2012) reported that myricetin as an α-amylase inhibitor was tightly bound and interacted with the critical amino acid residues including Glu233 and Asp300 in the active region of the enzyme *via* hydrogen bond and hydrophobic interactions. In the case of compound P2, which is a β-marsdenin glycoside, the additional sugar moiety bonded to the alcoholic group at C-3 of the aglycone structure donated a hydrogen to form an important conventional hydrogen bond with a bond length of 1.97 Å with Asp197. Given the role of Asp197 as a nucleophile, the interaction with P2 may disrupt the nucleophilic attack of Asp197 on polymeric substrates such as starch during the hydrolytic activity of the enzyme. An additional hydrogen bond was made between this sugar moiety with the side chain of Thr163 and by the steroidal framework of P2. Several pi-sigma, alkyl, and pi-alkyl hydrophobic interactions were made by the compounds with the active site residues of HPA to stabilize the complex. The additional tigloyl substituent attached at C-11 and C-12 in the steroidal framework of P2 also made a pi-alkyl hydrophobic interaction with Tyr151. In the cases of P3 and P4, hydroxyl groups on the pregnane structure conducted hydrogen bonds with amino acids such as Asp300, Glu233, Glu240, and Tyr163. Several alkyl and pi-alkyl hydrophobic interactions contributed to the stability of these compounds in the active site of HPA. In the same vein, the active site residues of HSA conducted similar interactions with Asp300 and Glu233 as well as other critical amino acids that define the active site of the enzyme.

Binding and interactions of an enzyme with its substrate and/or specific inhibitor is a dynamic process often assessed through MD simulations. When integrated with molecular docking simulation, MD simulations had a predictive capability of providing detailed insights into biomolecular structure and dynamics, which are often difficult to obtain from experimental procedures (Borhani and Shaw, 2012; Gyebi et al., 2021; Ogunyemi et al., 2022). Thus, it has been dubbed a reliable method for further validation of results obtained from molecular docking analysis. In the current study, the structural and conformational stability of the amylase-pregnane complexes were assessed through a 100-ns full atomistic MD simulation alongside the unbound enzyme (apoenzyme). Upon equilibration and stabilization, the RMSD values of the apo-HPA and the ligand–HPA complexes were maintained within 2 Å as depicted in Figure 8A. The observed initial high deviations may indicate major conformational transitions to attain stable conformation of the protein systems. Maintenance of RoG of the apoprotein and the pregnane–HPA complexes within 2 Å throughout the simulation period suggests a low tendency of an unfolding deviation from the original structure (Figure 8B). The surface accessible surface area (SASA), which estimates the level of the solvent accessibility surface of the protein systems also indicated the stability of the pregnane–HPA complexes (Figure 8C). The high number of hydrogen bonds detected in the pregnane–HPA complexes as depicted in Figure 8D further revealed the stability of the complexes. The steady SASA and RoG flow maintained throughout the simulation time is an indication of highly compacted and well-folded pregnane–HPA complexes with stable intermolecular bonds (Sinha and Wang, 2020; Ogunyemi et al., 2021; Olawale et al., 2022). Hydrogen bonds are important strong non-covalent interactions in protein–ligand complexes where they are responsible for the flexibility of the protein’s residues, maintaining a compact structure as well as a proper configuration (Gyebi et al., 2020; Gyebi et al., 2022). Taken together, the RMSD, SASA, RoG, and H-bond values revealed that the inhibitor–enzyme complexes were stable during the simulation and not likely to undergo an unfolding process. The pivotal roles of the amino acid residues that define the active site of a protein in attaining stable conformations with specific ligands can be assessed by computing the root mean square fluctuation (RMSF) from the MDS trajectory files of the biomolecular systems. The resulting plots indicate the flexibility attained by every residue of the protein in the dynamic environment. Thus, the greater the RMSF of amino acid residues in a protein motif, the greater the tendency of the subdomain to interact with the ligand. Important loop regions such as loop 3 (residues 105–111), loop 6 (residues 235–243), loop 7 (residues 295–315), and 8 (residues 346–353) are known to play important roles in the catalytic activity of HPA and are often targeted with inhibitors (Ann MacGregor, 1996; Borah et al., 2019). The highest RMSF values exhibited by the amino acids within loops 6, 7, and 8 of the HPA–P3 complex indicate a greater interaction tendency of the loops with the compound P3.

The maintenance of enzyme inhibitory activity of the pregnanes observed *in vitro* was assessed *in vivo* through oral administration of starch to albino rats. Peak blood glucose (PBG) and area under the curve (AUC) were significantly reduced in the albino rats that were orally administered with the 20 mg/kg dose of marsectohexol after starch loading. The ability of pregnane to lower the blood glucose was on par with acarbose in starch-fed albino rats. These results demonstrated the glucose-lowering effect of the marsectohexol possibly due to the alpha-amylase inhibitory activities observed *in vitro* and strong interactions with the enzyme catalytic residues observed *in silico*.

## Conclusion

There is an increasing interest among researchers to identify safer and more effective α-amylase inhibitors from antidiabetic food herbs and medicinal plants. In this study, we demonstrated the α-amylase inhibitory activity of a pregnane alongside three 17β-marsdenin pregnane glycosides, viz: iloneoside, 3-O-[6-deoxy-3-O-methyl-β-D-allopyranosyl-(1→4)-β-D-oleandropyranosyl]-17β-marsdenin, and 3-O-[6-deoxy-3-O-methyl-β-D-allopyranosyl-(1→4)-β-D-canaropyranosyl]-17β-marsdenin derived from *G. latifolium* using *in vitro*, *in silico*, and *in vivo* approaches. The pregnane-rich fractions and the SPP pregnanes exhibited inhibitory activity against porcine amylase *in vitro*. Molecular docking predicted strong affinity binding of these pregnanes with the porcine pancreatic alpha-amylase, human pancreatic amylase, and human salivary amylase. The binding interactions featured an array of hydrogen bonds and hydrophobic interactions, with critical catalytic residues involved in the acid/base catalysis of the target enzymes. These interactions were preserved in a dynamic environment as indicated by the computed root mean square deviation, radius of gyration, surface accessible surface area, and number of hydrogen bonds from the molecular dynamics trajectory files of pregnane–amylase complexes. The amylase also showed flexibility and interaction potential of its active site residues toward the compounds as indicated by the root mean square fluctuation. Furthermore, the P1 had significant reducing effects on the peak blood glucose and area under the curve in albino rats that were orally challenged with starch. Therefore, the SPPs derived from *G. latifolium* may be recommended as inhibitors of α-amylase, a diabetes target. However, experimental biophysical binding analysis and further *in vivo* experiments applied to disease conditions would be needed to validate the activity of these compounds toward their optimization, development, and exploitation as nutraceuticals and anti-diabetic drugs.

## Data Availability

The original contributions presented in the study are included in the article/Supplementary Material, further inquiries can be directed to the corresponding authors.

## References

[B1] Abdel-sattar E. El-Maraghy S. Dine R. Rizk S. (2016). Russelioside B, a pregnane glycoside ameliorates hyperglycemia in streptozotocin induced diabetic rats by regulating key enzymes of glucose metabolism. Chem. Biol. Interact. 252, 47–53. 10.1016/j.cbi.2016.03.033 27038876

[B2] Abdel-Sattar E. Harraz F. M. Ghareib S. A. Elberry A. A. Gabr S. Suliaman M. I. (2011). Antihyperglycaemic and hypolipidaemic effects of the methanolic extract of *Caralluma tuberculata* in streptozotocin-induced diabetic rats. Nat. Prod. Res. 25, 1171–1179. 10.1080/14786419.2010.490782 21740282

[B3] Abdel-Sattar E. Mehanna E. T. El-Ghaiesh S. H. Mohammad H. M. F. Elgendy H. A. Zaitone S. A. (2018). Pharmacological action of a pregnane glycoside, russelioside B, in dietary obese rats: Impact on weight gain and energy expenditure. Front. Pharmacol. 9, 990. 10.3389/fphar.2018.00990 30214412 PMC6125411

[B4] Abraham M. J. Murtola T. Schulz R. Páll S. Smith J. C. Hess B. (2015). Gromacs: High performance molecular simulations through multi-level parallelism from laptops to supercomputers. SoftwareX 1, 19–25. 10.1016/j.softx.2015.06.001

[B5] Akah P. Samuel U. Okolo C. E. (2011). Antidiabetic activity of aqueous and methanol extract and fractions of *Gongronema latifolium* (asclepidaceae) leaves in alloxan diabetic rats. J. Appl. Pharm. Sci. 1, 99–102.

[B6] Al-Hindi B. Yusoff N. A. Ahmad M. Atangwho I. J. Asmawi M. Z. Al-Mansoub M. A. (2019). Safety assessment of the ethanolic extract of *Gongronema latifolium* Benth. Leaves: a 90-day oral toxicity study in sprague dawley rats. BMC Complement. Altern. Med. 19, 152. 10.1186/s12906-019-2573-x 31253153 PMC6599297

[B7] Al-Hindi B. Yusoff N. A. Atangwho I. J. Ahmad M. Asmawi M. Z. Yam M. F. (2016). A soxhlet extract of *Gongronema latifolium* retains moderate blood glucose lowering effect and produces structural recovery in the pancreas of STZ-induced diabetic rats. Med. Sci. 4, 9. 10.3390/medsci4020009 PMC5635778 29083373

[B8] Alqahtani A. S. Hidayathulla S. Rehman M. T. ElGamal A. A. Al-Massarani S. Razmovski-Naumovski V. (2019). Alpha-amylase and alpha-glucosidase enzyme inhibition and antioxidant potential of 3-oxolupenal and katononic acid isolated from nuxia oppositifolia. Biomolecules 10, E61. 10.3390/biom10010061 31905962 PMC7022278

[B9] American Diabetes Association (2014). Diagnosis and classification of diabetes mellitus. Diabetes Care 37 (1), S81–S90. 10.2337/dc14-s081 24357215

[B10] Ann MacGregor E. (1996). “Structure and activity of some starch-metabolising enzymes,” in Progress in biotechnology. Editors Park K-H. Robyt J. F. Choi Y-D. (Elsevier), 109–124. 10.2337%2Fdc10-S062.

[B11] Ardalani H. Hejazi Amiri F. Hadipanah A. Kongstad K. T. (2021). Potential antidiabetic phytochemicals in plant roots: a review of *in vivo* studies. J. Diabetes Metab. Disord. 20, 1837–1854. 10.1007/s40200-021-00853-9 34900828 PMC8630315

[B12] Aryangat A. V. Gerich J. E. (2010). Type 2 diabetes: Postprandial hyperglycemia and increased cardiovascular risk. Vasc. Health Risk Manag. 6, 145–155. 10.2147/vhrm.s8216 20448799 PMC2860446

[B13] Ashwini S. Anitha R. (2017). Antihyperglycemic activity of *Caralluma fimbriata*: an *in vitro* approach. Pharmacogn. Mag. 13, S499–S504. 10.4103/pm.pm_59_17 29142405 PMC5669088

[B14] Bekker H. Berendsen H. Dijkstra E. Achterop S. Vondrumen R. Vanderspoel D. (1993). “Gromacs-a parallel computer for molecular-dynamics simulations,” in 4th international conference on computational physics (PC 92) (World Scientific Publishing), 252–256.

[B15] Beseni B. K. Matsebatlela T. M. Bagla V. P. Njanje I. Poopedi K. Mbazima V. (2019). Potential antiglycation and hypoglycaemic effects of *Toona ciliata* M. Roem. And schkuhria pinnata lam. Thell. Crude extracts in differentiated C2C12 cells. Evid. Based. Complement. Altern. Med. 12, 5406862. 10.1155/2019/5406862 PMC6363240 30805018

[B16] Borah P. K. Sarkar A. Duary R. K. (2019). Water-soluble vitamins for controlling starch digestion: Conformational scrambling and inhibition mechanism of human pancreatic α-amylase by ascorbic acid and folic acid. Food Chem. 288, 395–404. 10.1016/j.foodchem.2019.03.022 30902310

[B17] Borhani D. W. Shaw D. E. (2012). The future of molecular dynamics simulations in drug discovery. J. Comput. Aided. Mol. Des. 26, 15–26. 10.1007/s10822-011-9517-y 22183577 PMC3268975

[B18] Brayer G. D. Luo Y. Withers S. G. (1995). The structure of human pancreatic alpha-amylase at 1.8 A resolution and comparisons with related enzymes. Protein Sci. 4, 1730–1742. 10.1002/pro.5560040908 8528071 PMC2143216

[B19] Buchwald P. (2020). A single unified model for fitting simple to complex receptor response data. Sci. Rep. 10, 13386. 10.1038/s41598-020-70220-w 32770075 PMC7414914

[B20] Buisson G. Duée E. Haser R. Payan F. (1987). Three dimensional structure of porcine pancreatic alpha-amylase at 2.9 A resolution. Role of calcium in structure and activity. EMBO J. 6, 3909–3916. 10.1002/j.1460-2075.1987.tb02731.x 3502087 PMC553868

[B21] Carrasco M. Alcaíno J. Cifuentes V. Baeza M. (2017). Purification and characterization of a novel cold adapted fungal glucoamylase. Microb. Cell Fact. 16, 75. 10.1186/s12934-017-0693-x 28464820 PMC5414198

[B22] Chime S. A. Onyishi I. V. Ugwoke P. U. Attama A. A. (2014). Evaluation of the properties of Gongronema latifolium in phospholipon 90H based solid lipid microparticles (SLMs): an antidiabetic study. J. Diet. Suppl. 11, 7–18. 10.3109/19390211.2013.859212 24409977

[B23] Cho N. H. Shaw J. E. Karuranga S. Huang Y. da Rocha Fernandes J. D. Ohlrogge A. W. (2018). IDF Diabetes Atlas: Global estimates of diabetes prevalence for 2017 and projections for 2045. Diabetes Res. Clin. Pract. 138, 271–281. 10.1016/j.diabres.2018.02.023 29496507

[B24] Dandekar P. D. Kotmale A. S. Chavan S. R. Kadlag P. P. Sawant S. V. Dhavale D. D. (2021). Insights into the inhibition mechanism of human pancreatic α-amylase, a type 2 diabetes target, by dehydrodieugenol B isolated from ocimum tenuiflorum. ACS omega 6, 1780–1786. 10.1021/acsomega.0c00617 33521419 PMC7841778

[B25] Dhital S. Lin A. H. Hamaker B. R. Gidley M. J. Muniandy A. (2013). Mammalian mucosal alpha-glucosidases coordinate with alpha-amylase in the initial starch hydrolysis stage to have a role in starch digestion beyond glucogenesis. PLOS ONE 8, e62546. 10.1371/journal.pone.0062546 23638112 PMC3636141

[B26] Edet E. Akpanabiatu M. E Eno A. B Umoh I. Itam E. (2009). Effect of Gongronema latifolium crude leaf extract on some cardiac enzymes of alloxan-induced diabetic rats. Afr. J. Biochem. Res. 3, 366–369.

[B27] Elekofehinti O. O. Iwaloye O. Josiah S. S. Lawal A. O. Akinjiyan M. O. Ariyo E. O. (2021). Molecular docking studies, molecular dynamics and ADME/tox reveal therapeutic potentials of STOCK1N-69160 against papain-like protease of SARS-CoV-2. Mol. Divers. 25, 1761–1773. 10.1007/s11030-020-10151-w 33201386 PMC7670485

[B28] Etsassala N. G. E. R. Badmus J. A. Marnewick J. L. Iwuoha E. I. Nchu F. Hussein A. A. (2020). Alpha-glucosidase and alpha-amylase inhibitory activities, molecular docking, and antioxidant capacities of salvia aurita constituents. Antioxidants (Basel, Switz. 9, 1149. 10.3390/antiox9111149 PMC7699461 33228164

[B29] Gyebi G. A. Adebayo J. O. Olorundare O. E. Pardede A. Ninomiya M. Saheed A. O. (2018). Iloneoside: a cytotoxic ditigloylated pregnane glycoside from the leaves of Gongronema latifolium Benth. Nat. Prod. Res. 32, 2882–2886. 10.1080/14786419.2017.1385019 29034743

[B30] Gyebi G. A. Adegunloye A. P. Ibrahim I. M. Ogunyemi O. M. Afolabi S. O. Ogunro O. B. (2020). Prevention of SARS-CoV-2 cell entry: Insight from *in silico* interaction of drug-like alkaloids with spike glycoprotein, human ACE2, and TMPRSS2. J. Biomol. Struct. Dyn. 40, 2121–2145. 10.1080/07391102.2020.1835726 33089728 PMC7594191

[B31] Gyebi G. A. Elfiky A. A. Ogunyemi O. M. Ibrahim I. M. Adegunloye A. P. Adebayo J. O. (2021). Structure-based virtual screening suggests inhibitors of 3-Chymotrypsin-Like Protease of SARS-CoV-2 from Vernonia amygdalina and Occinum gratissimum. Comput. Biol. Med. 136, 104671. 10.1016/j.compbiomed.2021.104671 34332348 PMC8294106

[B32] Gyebi G. A. Ogunyemi O. M. Adefolalu A. A. Rodríguez-Martínez A. López-Pastor J. F. Banegas-Luna A. J. (2022). African derived phytocompounds may interfere with SARS-CoV-2 RNA capping machinery via inhibition of 2′-O-ribose methyltransferase: an *in silico* perspective. J. Mol. Struct. 1262, 133019. 10.1016/j.molstruc.2022.133019 35431328 PMC9002684

[B33] Habibuddin M. Daghriri H. A. Humaira T. Al Qahtani M. S. Hefzi A. A. (2008). Antidiabetic effect of alcoholic extract of Caralluma sinaica L. on streptozotocin-induced diabetic rabbits. J. Ethnopharmacol. 117, 215–220. 10.1016/j.jep.2008.01.021 18359177

[B34] Humphrey W. Dalke A. Schulten K. (1996). Vmd: Visual molecular dynamics. J. Mol. Graph. 14, 33–38. 10.1016/0263-7855(96)00018-5 8744570

[B35] Iwaloye O. Elekofehinti O. O. Kikiowo B. Oluwarotimi A. E. Fadipe M. T. (2021). Machine learning-based virtual screening strategy RevealsSome natural compounds as potential PAK4 inhibitors in triple negative breast cancer. Curr. Proteomics 18, 753–769. 10.2174/1570164618999201223092209

[B36] Kaur N. Kumar V. Nayak S. K. Wadhwa P. Kaur P. Sahu S. K. (2021). Alpha-amylase as molecular target for treatment of diabetes mellitus: a comprehensive review. Chem. Biol. Drug Des. 98, 539–560. 10.1111/cbdd.13909 34173346

[B37] Kiem P. V. Yen D. T. H. Hung N. V. Nhiem N. X. Tai B. H. Trang D. T. (2020). Five new pregnane glycosides from *Gymnema sylvestre* and their α-glucosidase and α-amylase inhibitory activities. Molecules 25, 2525. 10.3390/molecules25112525 PMC7321224 32481737

[B38] Kim Y. M. Jeong Y. K. Wang M. H. Lee W. Y. Rhee H. I. (2005). Inhibitory effect of pine extract on alpha-glucosidase activity and postprandial hyperglycemia. Nutrition 21, 756–761. 10.1016/j.nut.2004.10.014 15925302

[B39] Lankatillake C. Luo S. Flavel M. Lenon G. B. Gill H. Huynh T. (2021). Screening natural product extracts for potential enzyme inhibitors: Protocols, and the standardisation of the usage of blanks in α-amylase, α-glucosidase and lipase assays. Plant Methods 17, 3. 10.1186/s13007-020-00702-5 33407662 PMC7789656

[B40] Liu S. Chen Z. Wu J. Wang L. Wang H. Zhao W. (2013). Appetite suppressing pregnane glycosides from the roots of *Cynanchum auriculatum* . Phytochemistry 93, 144–153. 10.1016/j.phytochem.2013.03.010 23602053

[B41] Mahomoodally F. M. Subratty A. H. Gurib-Fakim A. Choudhary M. I. (2012). Antioxidant, antiglycation and cytotoxicity evaluation of selected medicinal plants of the Mascarene Islands. BMC Complement. Altern. Med. 12, 165. 10.1186/1472-6882-12-165 23020844 PMC3517752

[B42] Malviya N. Jain S. Malviya S. (2010). Antidiabetic potential of medicinal plants. Acta Pol. Pharm. 67, 113–118. PMID: 20369787. 20369787

[B43] Miller G. L. (1959). Use of dinitrosalicylic acid reagent for determination of reducing sugar. Anal. Chem. 31, 426–428. 10.1021/ac60147a030

[B44] Mohamed E. A. Siddiqui M. J. Ang L. F. Sadikun A. Chan S. H. Tan S. C. (2012). Potent α-glucosidase and α-amylase inhibitory activities of standardized 50% ethanolic extracts and sinensetin from *Orthosiphon stamineus* Benth as anti-diabetic mechanism. BMC Complement. Altern. Med. 12, 176. 10.1186/1472-6882-12-176 23039079 PMC3533584

[B45] Moorthy N. S. Ramos M. J. Fernandes P. A. (2012). Studies on alpha-glucosidase inhibitors development: Magic molecules for the treatment of carbohydrate mediated diseases. Mini Rev. Med. Chem. 12, 713–720. 10.2174/138955712801264837 22512574

[B46] Morris G. M. Huey R. Lindstrom W. Sanner M. F. Belew R. K. Goodsell D. S. (2009). AutoDock4 and AutoDockTools4: automated docking with selective receptor flexibility. J. Comput. Chem. 30, 2785–2791. 10.1002/jcc.21256 19399780 PMC2760638

[B47] Nahoum V. Roux G. Anton V. Rougé P. Puigserver A. Bischoff H. (2000). Crystal structures of human pancreatic alpha-amylase in complex with carbohydrate and proteinaceous inhibitors. Biochem. J. 346 (1), 201–208. 10.1042/bj3460201 10657258 PMC1220841

[B48] Nyambe-Silavwe H. Villa-Rodriguez J. A. Ifie I. Holmes M. Aydin E. Jensen J. M. (2015). Inhibition of human α-amylase by dietary polyphenols. J. Funct. Foods 19, 723–732. 10.1016/j.jff.2015.10.003

[B49] O'Boyle N. M. Banck M. James C. A. Morley C. Vandermeersch T. Hutchison G. R. (2011). Open Babel: an open chemical toolbox. J. Cheminform. 3, 33. 10.1186/1758-2946-3-33 21982300 PMC3198950

[B50] Ogunyemi O. M. Gyebi A. G. Adebayo J. O. Oguntola J. A. Olaiya C. O. (2020). Marsectohexol and other pregnane phytochemicals derived from Gongronema latifolium as α-amylase and α-glucosidase inhibitors: *In vitro* and molecular docking studies. SN Appl. Sci. 2, 2119. 10.1007/s42452-020-03951-0

[B51] Ogunyemi O. M. Gyebi G. A. Ibrahim I. M. Esan A. M. Olaiya C. O. Soliman M. M. (2022). Identification of promising multi-targeting inhibitors of obesity from Vernonia amygdalina through computational analysis. Mol. Divers. 10.1007/s11030-022-10397-6 35179699

[B52] Ogunyemi O. M. Gyebi G. A. Ibrahim I. M. Olaiya C. O. Ocheje J. O. Fabusiwa M. M. (2021). Dietary stigmastane-type saponins as promising dual-target directed inhibitors of SARS-CoV-2 proteases: a structure-based screening. RSC Adv. 11, 33380–33398. 10.1039/D1RA05976A 35497510 PMC9042289

[B53] Olawale F. Iwaloye O. Olofinsan K. Ogunyemi O. M. Gyebi G. A. Ibrahim I. M. (2022). Homology modelling, vHTS, pharmacophore, molecular docking and molecular dynamics studies for the identification of natural compound-derived inhibitor of MRP3 in acute leukaemia treatment. Chem. Pap. 76, 3729–3757. 10.1007/s11696-022-02128-w

[B54] Olubomehin O. O. Abo K. A. Ajaiyeoba E. O. (2013). Alpha-amylase inhibitory activity of two Anthocleista species and *in vivo* rat model anti-diabetic activities of Anthocleista djalonensis extracts and fractions. J. Ethnopharmacol. 146, 811–814. 10.1016/j.jep.2013.02.007 23422334

[B55] Oostenbrink C. Villa A. Mark A. E. Van Gunsteren W. F. (2004). A biomolecular force field based on the free enthalpy of hydration and solvation: The GROMOS force-field parameter sets 53A5 and 53A6. J. Comput. Chem. 25, 1656–1676. 10.1002/jcc.20090 15264259

[B56] Perez-Gutierrez R. M. Damian-Guzman M. (2012). Meliacinolin: a potent α-glucosidase and α-amylase inhibitor isolated from Azadirachta indica leaves and *in vivo* antidiabetic property in streptozotocin-nicotinamide-induced type 2 diabetes in mice. Biol. Pharm. Bull. 35, 1516–1524. 10.1248/bpb.b12-00246 22975503

[B57] Ponnusamy S. Zinjarde S. Bhargava S. Rajamohanan P. R. Ravikumar A. (2012). Discovering Bisdemethoxycurcumin from Curcuma longa rhizome as a potent small molecule inhibitor of human pancreatic α-amylase, a target for type-2 diabetes. Food Chem. 135, 2638–2642. 10.1016/j.foodchem.2012.06.110 22980852

[B58] Poovitha S. Parani M. (2016). *In vitro* and *in vivo* α-amylase and α-glucosidase inhibiting activities of the protein extracts from two varieties of bitter gourd (Momordica charantia L.). BMC Complement. Altern. Med. 16 (1), 185. 10.1186/s12906-016-1085-1 27454418 PMC4959359

[B59] Riaz Z. Ali M. Qureshi Z. Mohsin M. (2020). *In vitro* investigation and evaluation of novel drug based on polyherbal extract against type 2 diabetes. J. Diabetes Res. 2020, 1–9. 10.1155/2020/7357482

[B60] Rouzbehan S. Moein S. Homaei A. Moein M. R. (2017). Kinetics of alpha-glucosidase inhibition by different fractions of three species of *labiatae* extracts: a new diabetes treatment model. Pharm. Biol. 55, 1483–1488. 10.1080/13880209.2017.1306569 28367665 PMC7011978

[B61] Rydberg E. H. Li C. Maurus R. Overall C. M. Brayer G. D. Withers S. G. (2002). Mechanistic analyses of catalysis in human pancreatic alpha-amylase: Detailed kinetic and structural studies of mutants of three conserved carboxylic acids. Biochemistry 41, 4492–4502. 10.1021/bi011821z 11914097

[B62] Saini P. Debnath P. Tuli H. S. Kashyap D. (2017). Recent advances in molecular docking studies using α-glucosidase inhibitors. J. Biol. Chem. Sci. 4, 304–308.

[B63] Saini P. Sharma A. Tuli H. S. Kashyap D. Kumar Mishra N. Debnath P. (2016). *In silico* comparative analysis of natural metabolites to alpha glucosidase inhibitors. J. Biol. Chem. Sci. 3, 226–232.

[B64] Sales P. M. Souza P. M. Simeoni L. A. Silveira D. (2012). α-Amylase inhibitors: a review of raw material and isolated compounds from plant source. J. Pharm. Pharm. Sci. 15, 141–183. 10.18433/j35s3k 22365095

[B65] Schüttelkopf A. W. Van Aalten D. M. (2004). Prodrg: a tool for high-throughput crystallography of protein–ligand complexes. Acta Crystallogr. D. Biol. Crystallogr. 60, 1355–1363. 10.1107/s0907444904011679 15272157

[B66] Seigner C. Prodanov E. Marchis-Mouren G. (1987). The determination of subsite binding energies of porcine pancreatic alpha-amylase by comparing hydrolytic activity towards substrates. Biochim. Biophys. Acta 913, 200–209. 10.1016/0167-4838(87)90331-1 3496119

[B67] Singla K. R. Singh R. Dubey K. A. (2016). Important aspects of post-prandial antidiabetic drug, acarbose. Curr. Top. Med. Chem. 16, 2625–2633. 10.2174/1568026616666160414123500 27086787

[B68] Sinha S. Wang S. M. (2020). Classification of VUS and unclassified variants in BRCA1 BRCT repeats by molecular dynamics simulation. Comput. Struct. Biotechnol. J. 18, 723–736. 10.1016/j.csbj.2020.03.013 32257056 PMC7125325

[B69] Srisurichan S. Puthong S. Pornpakakul S. (2014). Pregnane-type steroidal glycosides from Gymnema griffithii Craib. Phytochemistry 106, 197–206. 10.1016/j.phytochem.2014.06.014 25053002

[B70] Stiefel D. J. Keller P. J. (1973). Preparation and some properties of human pancreatic amylase including a comparison with human parotid amylase. Biochim. Biophys. Acta 302, 345–361. 10.1016/0005-2744(73)90163-0 4699244

[B71] Trott O. Olson A. J. (2010). AutoDock Vina: Improving the speed and accuracy of docking with a new scoring function, efficient optimization, and multithreading. J. Comput. Chem. 31, 455–461. 10.1002/jcc.21334 19499576 PMC3041641

[B72] Unuofin J. Lebelo S. (2020). “Review article antioxidant effects and mechanisms of medicinal plants and their bioactive compounds for the prevention and treatment of type 2 diabetes: an updated review,” in Oxidative medicine and cellular longevity 2020. 10.1155/2020/1356893 PMC7042557 32148647

[B73] Venkatesh S. Reddy G. D. Reddy B. M. Ramesh M. Rao A. V. (2003). Antihyperglycemic activity of Caralluma attenuata. Fitoterapia 74, 274–279. 10.1016/s0367-326x(03)00021-2 12727493

[B74] Whitcomb D. C. Lowe M. E. (2007). Human pancreatic digestive enzymes. Dig. Dis. Sci. 52, 1–17. 10.1007/s10620-006-9589-z 17205399

[B75] Williams L. K. Li C. Withers S. G. Brayer G. D. (2012). Order and disorder: Differential structural impacts of myricetin and ethyl caffeate on human amylase, an antidiabetic target. J. Med. Chem. 55, 10177–10186. 10.1021/jm301273u 23050660

[B76] Zhang P. Zhang X. Brown J. Vistisen D. Sicree R. Shaw J. (2010). Global healthcare expenditure on diabetes for 2010 and 2030. Diabetes Res. Clin. Pract. 87, 293–301. 10.1016/j.diabres.2010.01.026 20171754

