# Supplementary material for: Inhibition mechanism of alpha-amylase, a diabetes target, by a steroidal pregnane and pregnane glycosides derived from Gongronema latifolium Benth
Source: Front Mol Biosci. 2022 Aug 10;9:866719. doi: 10.3389/fmolb.2022.866719 (PMC9399641; doi:10.3389/fmolb.2022.866719)
Supplement: Supplementary file 1 [file Table1.DOCX]

Supplementary Material

# Supplementary Table

Table S1: Binding site coordinates of Human Pancreatic Amylase and Human Salivary Amylase

|  | 1OSE | | 4GQR | | 1SMD | |
| --- | --- | --- | --- | --- | --- | --- |
|  | Targeted | Blind | Targeted | Blind | Targeted | Blind |
| center_x | 35.97 | 34.02 | 9.38 | 7.27 | 9.04 | 7.21 |
| center_y | 36.04 | 31.50 | 21.83 | 27.53 | 51.78 | 61.33 |
| center_z | 8.23 | 15.48 | 45.81 | 49.03 | 19.09 | 19.23 |
| Size x | 20.52 | 41.37 | 25.0 | 40.33 | 25.0 | 45.68 |
| Size y | 22.62 | 59.24 | 26.0 | 54.89 | 22.15 | 65.47 |
| Size z | 17.84 | 41.43 | 25.0 | 30.89 | 25.0 | 48.85 |
